# An illustrated guide for characters and terminology used in descriptions of Phlebotominae (Diptera, Psychodidae)

**DOI:** 10.1051/parasite/2017027

**Published:** 2017-07-21

**Authors:** Eunice A. B. Galati, Fredy Galvis-Ovallos, Phillip Lawyer, Nicole Léger, Jérôme Depaquit

**Affiliations:** 1 Departamento de Epidemiologia, Faculdade de Saúde Pública, Universidade de São Paulo Av. Dr. Arnaldo 715 01246-904 São Paulo Brazil; 2 Laboratory of Parasitic Diseases, National Institute of Allergy and Infectious Diseases, National Institutes of Health Bethesda MD 20892 USA; 3 Monte L. Bean Life Science Museum, Brigham Young University Provo UT 84602 USA; 4 Université de Reims Champagne-Ardenne, ANSES, SFR Cap Santé, EA4688-USC “Transmission vectorielle et épidémiosurveillance de maladies parasitaires (VECPAR)” 51 rue Cognacq-Jay 51096 Reims Cedex France

**Keywords:** Phlebotominae, Taxonomy, Morphology, Species description

## Abstract

Phlebotomine (Diptera, Psychodidae, Phlebotominae) taxonomy has been studied extensively, primarily due to the role of these flies as vectors of various parasites, including species of *Leishmania*, *Bartonella* and arboviruses that cause diseases in humans and other vertebrates. We present some topics discussed at a round-table on phlebotomine taxonomy held at the Ninth International Symposium on Phlebotomine Sandflies (ISOPS IX) in Reims, France, in June 2016. To date, approximately one thousand phlebotomine species have been described worldwide, although in varying languages and mostly without standardization of characters and terminology. In the interest of standardization, we list the characters that should minimally be considered in the description of new phlebotomine taxa as well as annotated illustrations of several characters. For these characters, multiple illustrations are provided to show some of the variations. The preferred terms for all pertinent characters are listed as well as their synonyms in English, Portuguese, and French. Finally, we offer an updated list of abbreviations to be used for generic and subgeneric names.

## Abstract (fr)

La taxonomie des phlébotomes (Diptera, Psychodidae, Phlebotominae) a été largement étudiée, principalement en raison du rôle de ces diptères comme vecteurs de divers parasites, y compris des espèces de *Leishmania*, *Bartonella* et des arbovirus qui causent des maladies chez l’homme et d’autres vertébrés. Nous présentons certains thèmes abordés lors d’une table ronde sur la taxonomie des phlébotomes tenue lors du 9^ème^ Symposium international sur les phlébotomes (ISOPS IX) à Reims, France, en juin 2016. À ce jour, environ mille espèces de phlébotomes ont été décrites dans le monde entier, bien que dans des langues variées et surtout sans standardisation des caractères et de la terminologie. Dans l’intérêt de la normalisation, nous énumérons les caractères qui devraient être considérés lors de la description de nouveaux taxons de phlébotomes et fournissons des illustrations légendées de nombre d’entre eux. Pour ces caractères, plusieurs illustrations sont fournies pour montrer une partie de la variabilité. Les termes préférés pour tous les caractères pertinents sont répertoriés ainsi que leurs synonymes en anglais, portugais et français. Enfin, nous proposons une liste actualisée des abréviations à utiliser pour les genres et sous-genres.

## Introduction

Phlebotomine (Diptera, Psychodidae, Phlebotominae) taxonomy has been studied extensively, primarily due to the role of these flies as vectors of various agents, including species of *Leishmania*, *Bartonella* and arboviruses that cause infections in humans and other vertebrates. To date, approximately one thousand phlebotomine species have been described worldwide, although in varying languages and mostly without standardization of characters and terminology. During the Ninth International Symposium on Phlebotomine Sandflies (ISOPS IX) held in Reims from June 28 to July 1, 2016 [4], a round-table on Systematics was co-chaired by EAB Galati, P Lawyer, N Léger, and J Depaquit. We report in this paper the results of discussions on the following topics: the use of the informal term sandflies (or sand flies); methods for permanent preservation and mounting of phlebotomine specimens and appropriate places for deposition of type specimens; a recommendation regarding terms used in morphological descriptions of sand flies and their synonyms, illustrated by several drawings with captions; the characters to be used in taxonomic discussion and to be drawn when a new species is described, and some comments about the systematics of phlebotomines and integrative taxonomy.

## On the use of the common name sand flies

Throughout history, there have been discussions on the use of the popular term sandflies (as one word) or sand flies (two words). Beyond the problem of its true form, this common name has also been applied to other dipterans (e.g. Ceratopogonidae and Simuliidae). Furthermore, some feel that the name does not seem appropriate to the habitat of Neotropical phlebotomine species, whereas others argue that the name was originally given to reflect the color rather than the habitat of these flies. Some colleagues suggested using the term “phlebotomine flies” to replace “sand flies” or “sandflies”; others suggested using “phlebotomine sand flies.” The discussion ended without achieving a consensus.

## On type specimens

In the past and even more recently, phlebotomine type specimens have been deposited in institutions or schools other than museums [1, 9, 10]. It is very important to deposit at least the holotype in a collection under the supervision of a curator; however, political or administrative changes in these institutions can affect the specimens’ preservation and availability under conditions suitable for examination. Consistent with Articles 16C and 72F of the fourth edition of the International Code of Zoological Nomenclature (ICZN), we strongly recommend that authors of species descriptions deposit type and voucher specimens in one or more institutions that maintain a research collection with proper facilities for preserving them and making them accessible for study. It is critical for every institution in which material is deposited to ensure that all name-bearing types are clearly marked and recognizable as such. Moreover, the repository must be capable of taking all necessary steps to preserve these specimens, make them accessible for study, publish lists of name-bearing types in their possession or custody and, in so far as possible, communicate upon request information concerning name-bearing types [6]. In addition to depositing type and voucher specimens, we recommend registration with Zoobank (http://www.zoobank.org) for all nomenclatural acts (published usages of scientific names of animals, which affect their nomenclature or the typification of a nominal taxon), publications of original taxonomic descriptions, and references to authors and designation of type specimens.

## Methods of preserving phlebotomine specimens

Many type specimens of phlebotomine species and other specimens in many reference collections were mounted in non-permanent media such as Hoyer’s Medium or Berlese and have long since deteriorated so that many of the diagnostic characters of the specimens are neither visible nor distinguishable, thereby rendering the specimen useless. It was proposed that a protocol using a permanent medium such as Canada balsam be adopted and standardized, in so far as possible, for preservations of all specimens that are to be deposited in museums for future reference.

## Suggested guidelines for the description of new phlebotomine species

**Figure 1.**
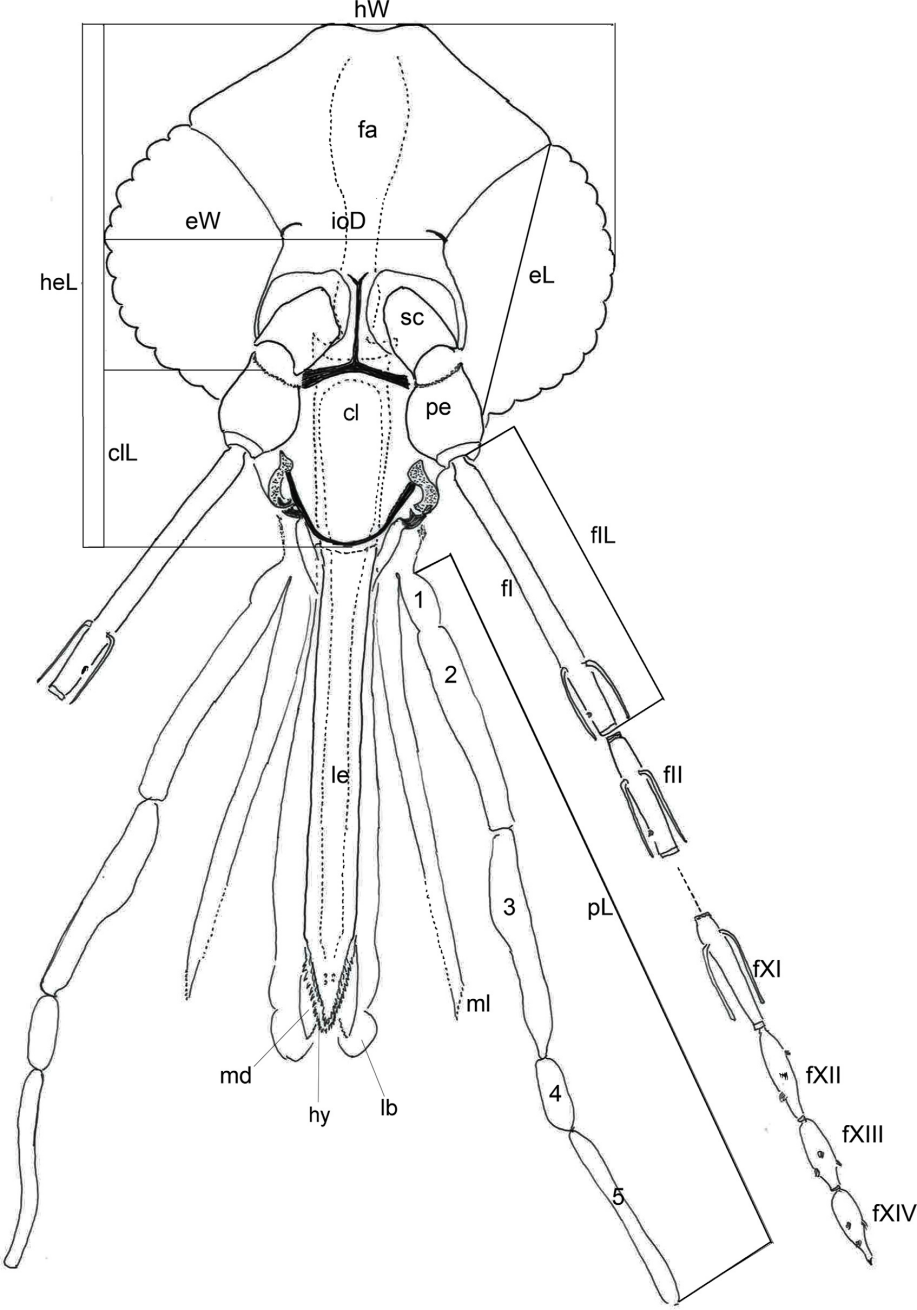
Dorsal view of the head and its appendages of a phlebotomine female: cl – clypeus; fI – 1st flagellomere; fII – 2nd flagellomere; hy – hypopharynx; ml – maxillary lacinia; lb – labium; le – labrum-epipharynx; md – mandible; pe – pedicel; pha – pharynx; p1 – 1st palpal segment; p2 – 2nd palpal segment; p3 – 3rd palpal segment; p4 – 4th palpal segment; p5 – 5th palpal segment; sc – scape; most frequently used measurements: eL – eye length; eW – eye width; clL – clypeus length; fIL – 1st flagellomere length; heL – head length; hW – head width; ioD – interocular distance; pL – palpus length – *Nyssomyia intermedia*.

**Figure 2.**
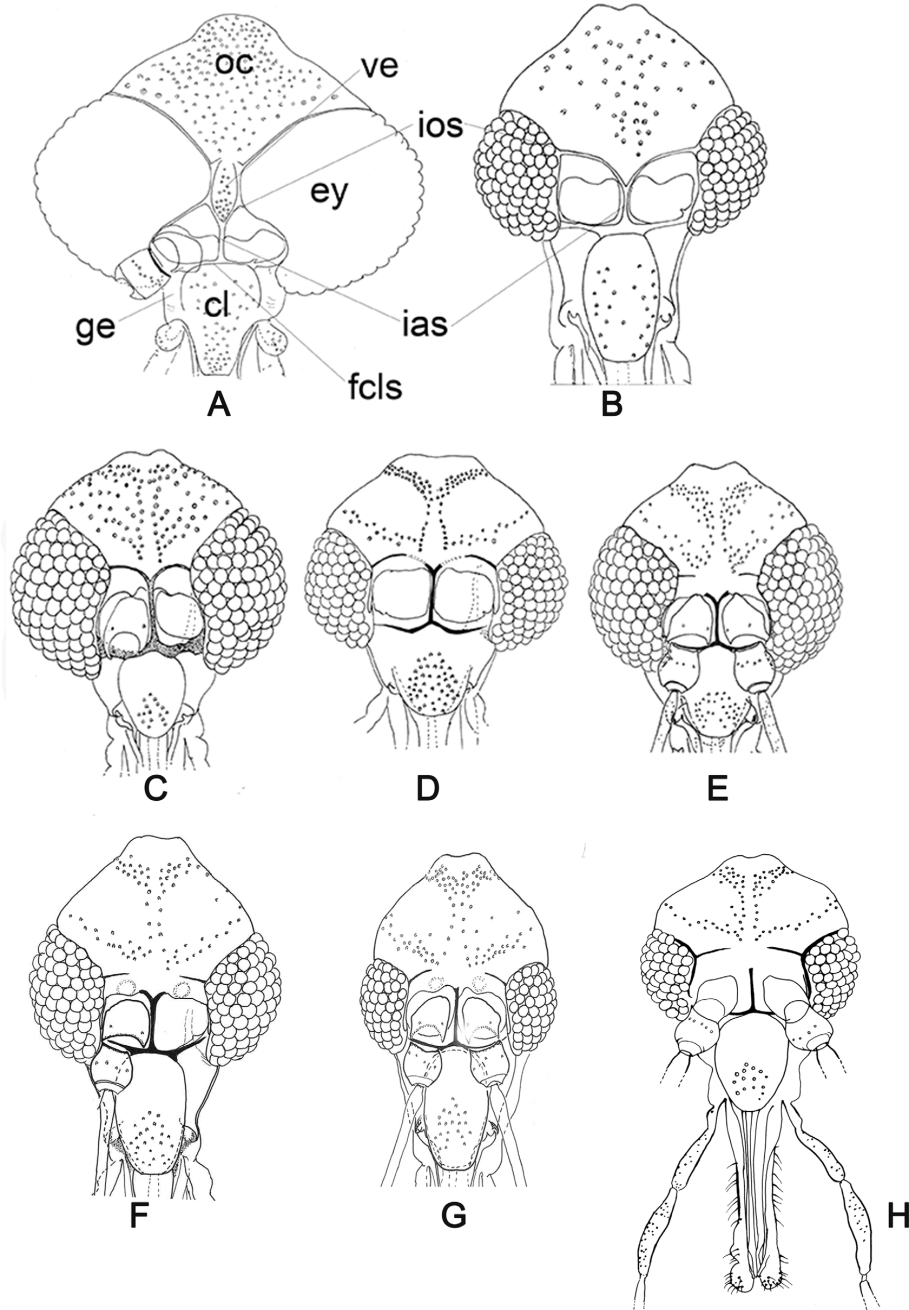
(A–G) Dorsal view of the head of Bruchomyiinae and Phlebotominae with the distribution of the setae on some sclerites, the relation of clypeus to eyes and aspects of some structures (A) Bruchomyiinae (*Bruchomyia sp.*). (B) Phlebotominae: *Warileya phlebotomanica*; (C) *Brumptomyia brumpti*; (D) *Sergentomyia (Sergentomyia) minuta*; (E) *Pintomyia (Pifanomyia) maranonensis*; (F) *Lutzomyia* (*Helcocyrtomyia*) *tejadai*; (G) *Lu.* (*Helcocyrtomyia*) *blancasi*; (H) *Phlebotomus* (*Euphlebotomus*) *barguesae*. ey – eye; fcls – frontoclypeal suture; ge – gena; ias – interantennal suture; ios – interocular suture; oc – occiput; ve – vertex.

**Figure 3.**
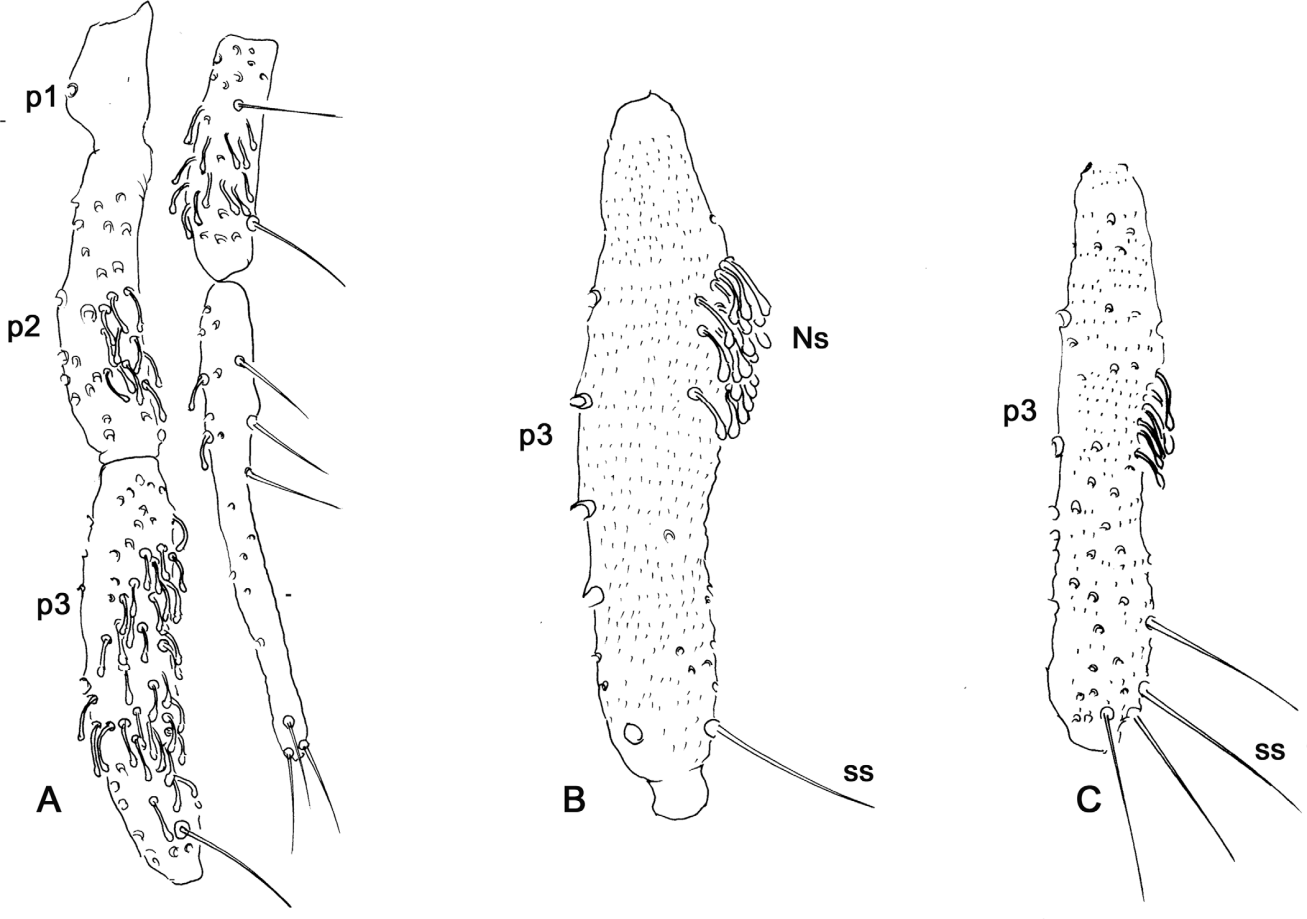
Palpus of Phlebotominae. (A) 1st–5th palpal segments of phlebotomines: Newstead’s sensilla (Ns) dispersed on p3 and present from 2nd to 5th segment – *Psathyromyia naftalekatzi.* (B) Newstead’s sensilla concentrated on basal part of the segment and only one simple seta (ss) – *Micropygomyia echinathopharynx*. (C) Several simple setae on p3 *– Warileya phlebotomanica.*

**Figure 4.**
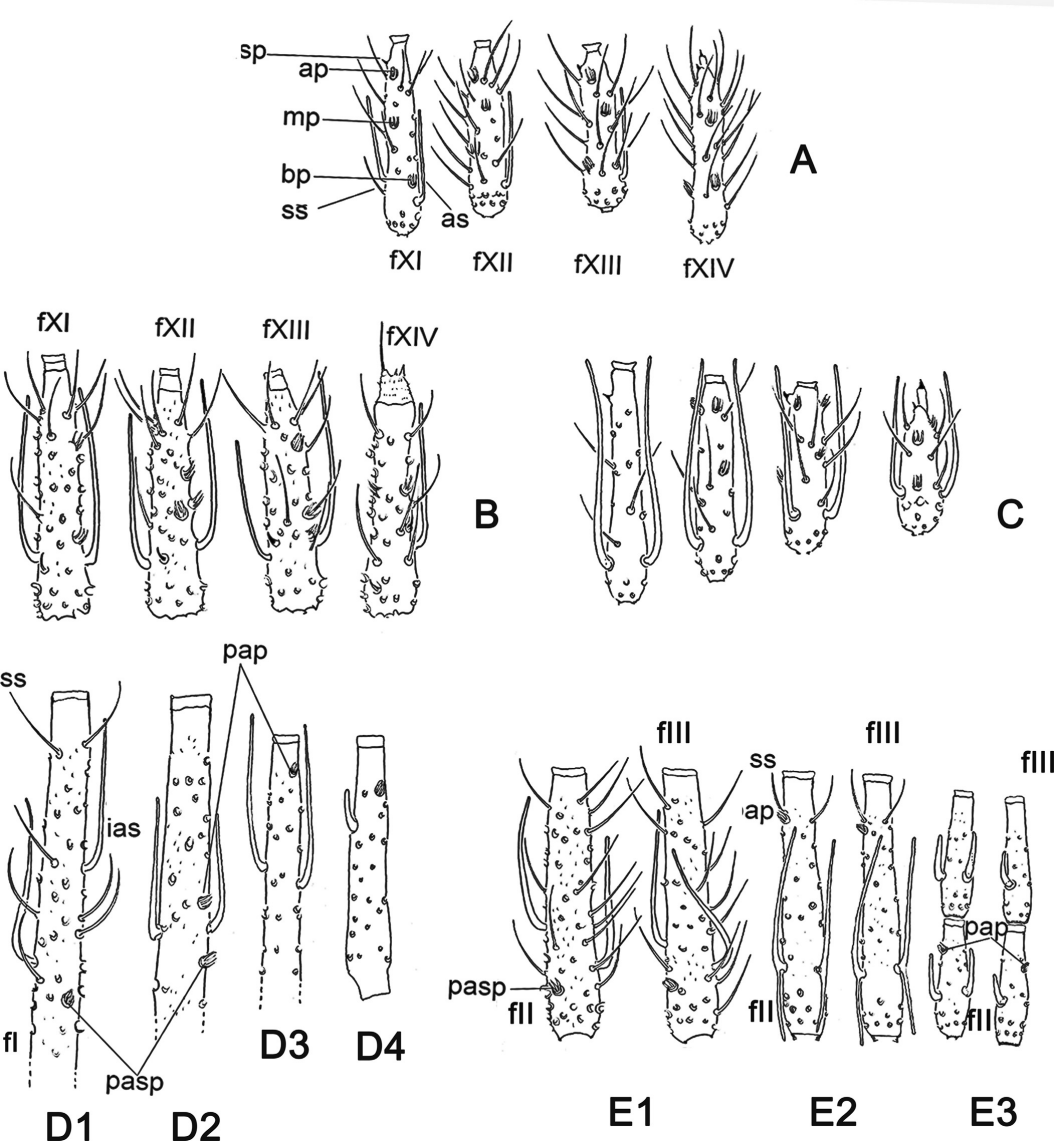
Aspects on the presence and distribution of ascoids, papillae, and simple setae on flagellomeres of Phlebotominae: (A–C) apical flagellomeres (fXI–fXIV): (A) *Evandromyia* (*Barrettomyia*) *tupynambai* (♂); (B) *Warileya rotundipennis* (♀); (C) *Trichophoromyia ubiquitalis* (♀). (D1–D4) First flagellomere (fI) of phlebotomine: (D1) *Warileya rotundipennis* (♀); (D2) *Psychodopygus squamiventris* (♀); (D3) *Evandromyia aroucki* (♂); (D4) *Sergentomyia dubia* (♂). (E1–E4) 2nd and 3rd flagellomeres of phlebotomines: (E1) *Warileya rotundipennis* (♀); (E2) *Psathyromyia shannoni* (♀); (E3) *Sergentomyia dubia* (♀); (E4) *Sergentomyia dubia* (♂). ap – Apical papilla; as – ascoid; bp – basal papilla; ias – internal ascoid; mp – median papilla; p – papilla; pap – preapical papilla; pasp – preascoidal papilla; sp – spiniform papilla; ss – simple seta.

**Figure 5.**
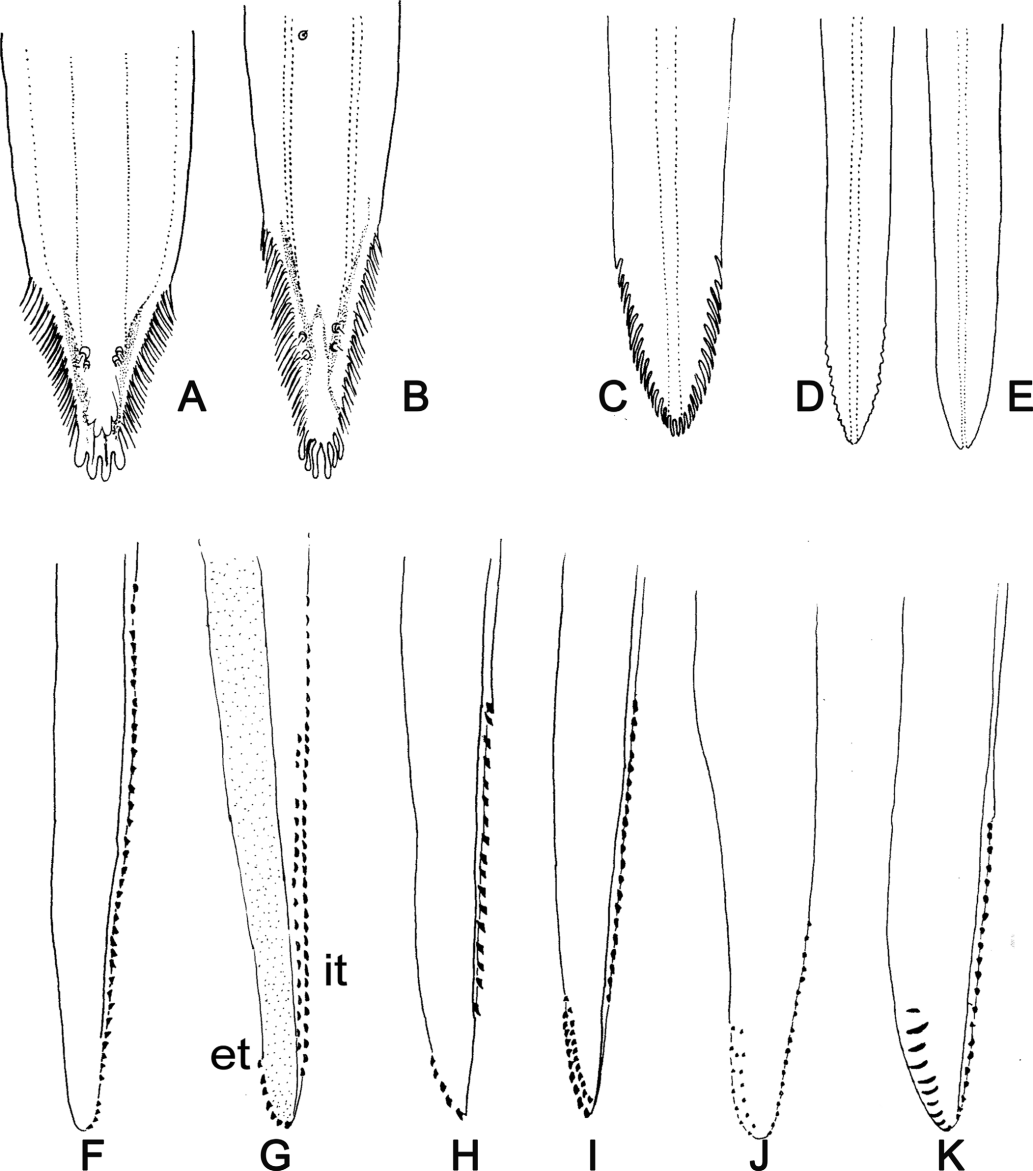
Mouth parts of Phlebotominae. (A, B) Apical region of the labrum-epipharynx of phlebotomine females: (A) *Micropygomyia vexator*; (B) *Lutzomyia longipalpis.* (C–E) Apical region of the hypopharynx of phlebotomine females: (C) *Lutzomyia longipalpis*; (D) *Sciopemyia sordellii*; (E) *Micropygomyia cayennensis.* (F–K) Maxillary lacinia of phlebotomine females: (F) *Warileya phlebotomanica*; (G) *Warileya rotundipennis*; (H) *Lutzomyia cruciata*; (I) *Nyssomyia intermedia*; (J) *Micropygomyia quinquefer*; (K) *Micropygomyia longipennis.* et – External teeth of maxillary lacinia; it – internal teeth of maxillary lacinia.

**Figure 6.**
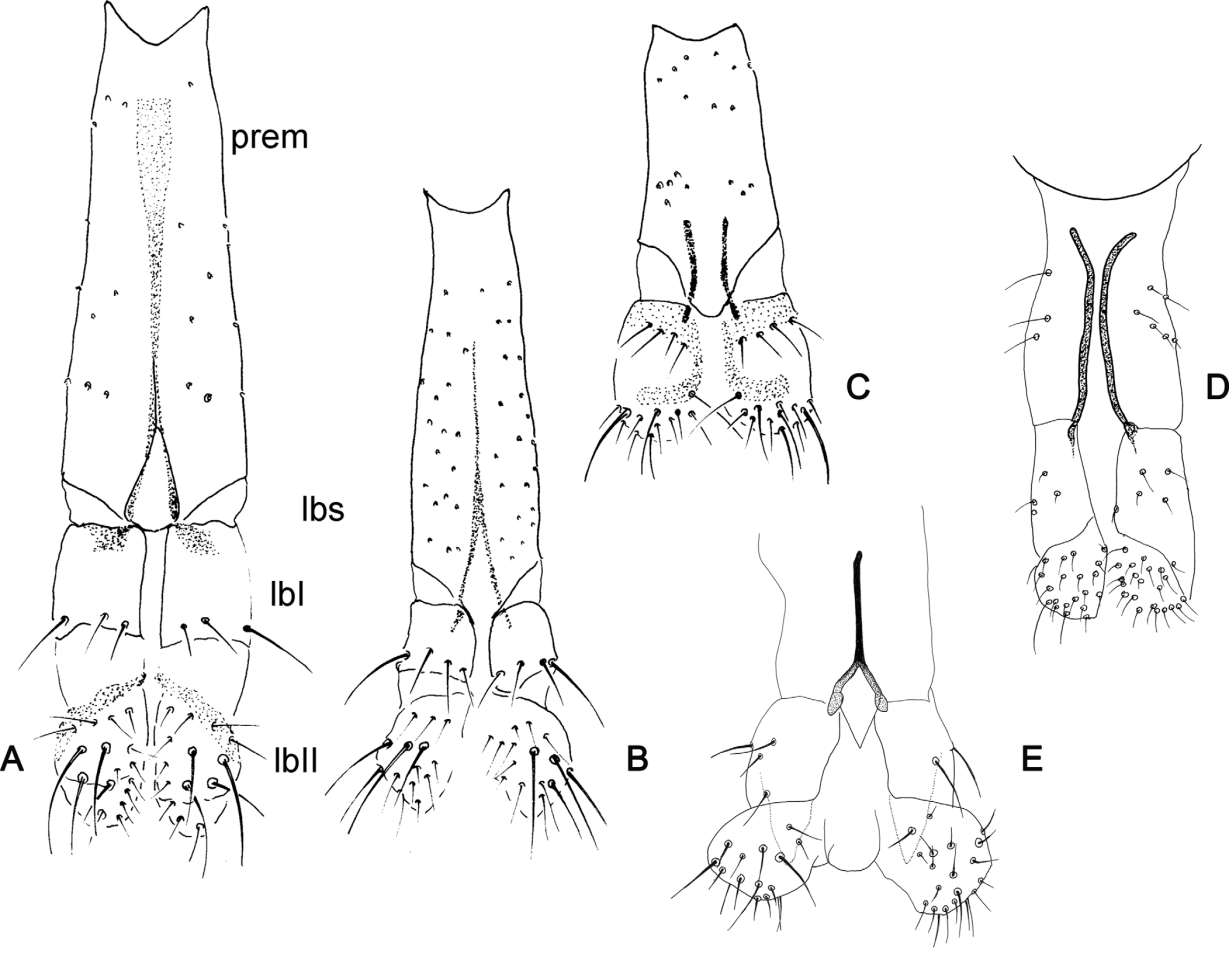
Labium of phlebotomine females in ventral view. (A) *Warileya phlebotomanica*; (B) *Lutzomyia amarali*; (C) *Sergentomyia minuta*; (D) *Idiophlebotomus padillarum*; (E) *Chinius eunicegalatiae.* lbI – Labellum I; lbII – labellum II; lbs – labial suture; prem – prementum.

**Figure 7.**
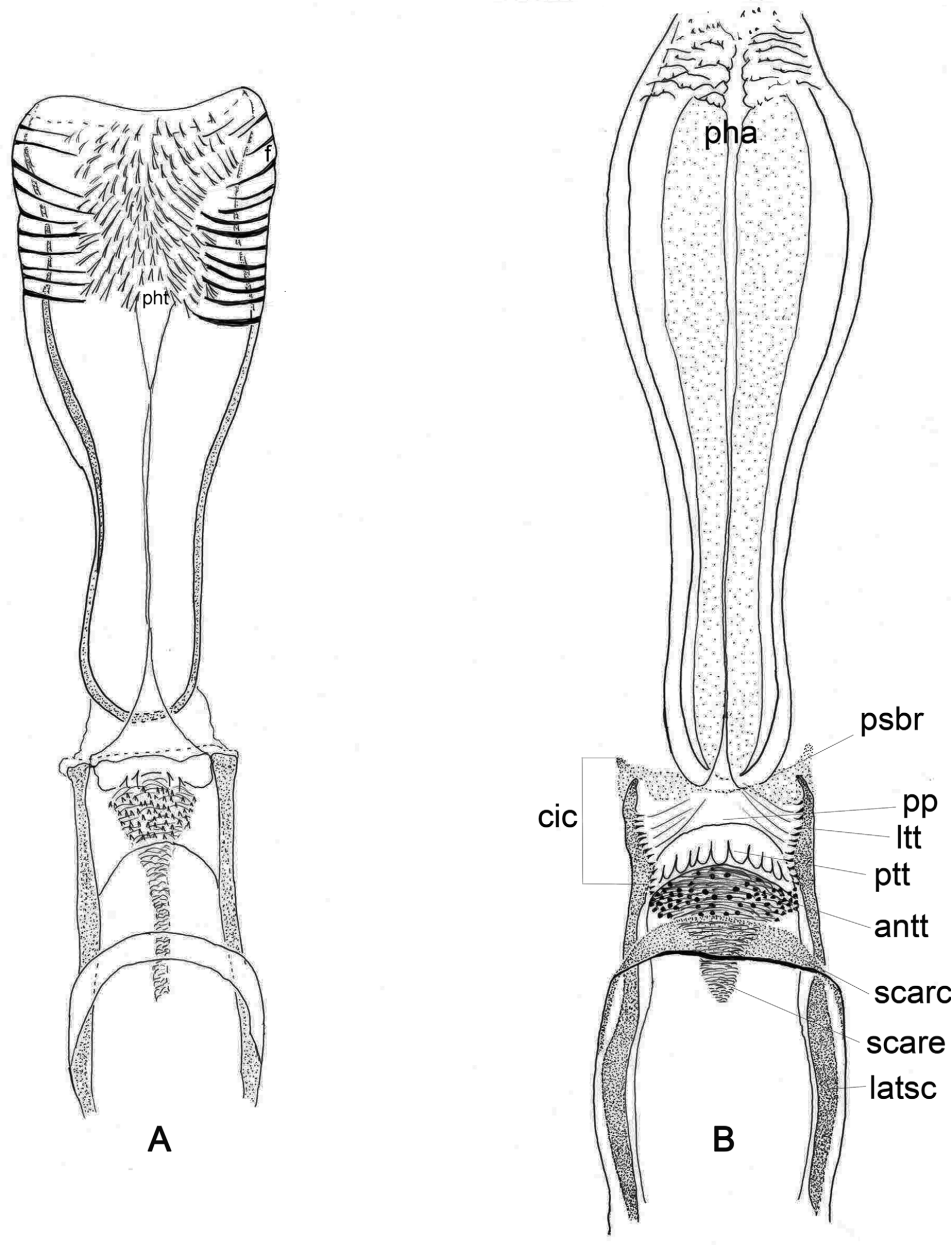
Cibarium and pharynx of phlebotomine females. (A) *Micropygomyia atroclavata*; (B) *Bichromomyia flaviscutellata*. antt – Anterior teeth; cic – cibarial chamber; f – fold; ltt – lateral teeth; pha – pharynx; pht – pharyngeal teeth; psbr – posterior bridge; ptt – posterior teeth; pp – posterior protuberance; latsc – lateral sclerite; scarc – sclerotized arch; scare – sclerotized area.

**Figure 8.**
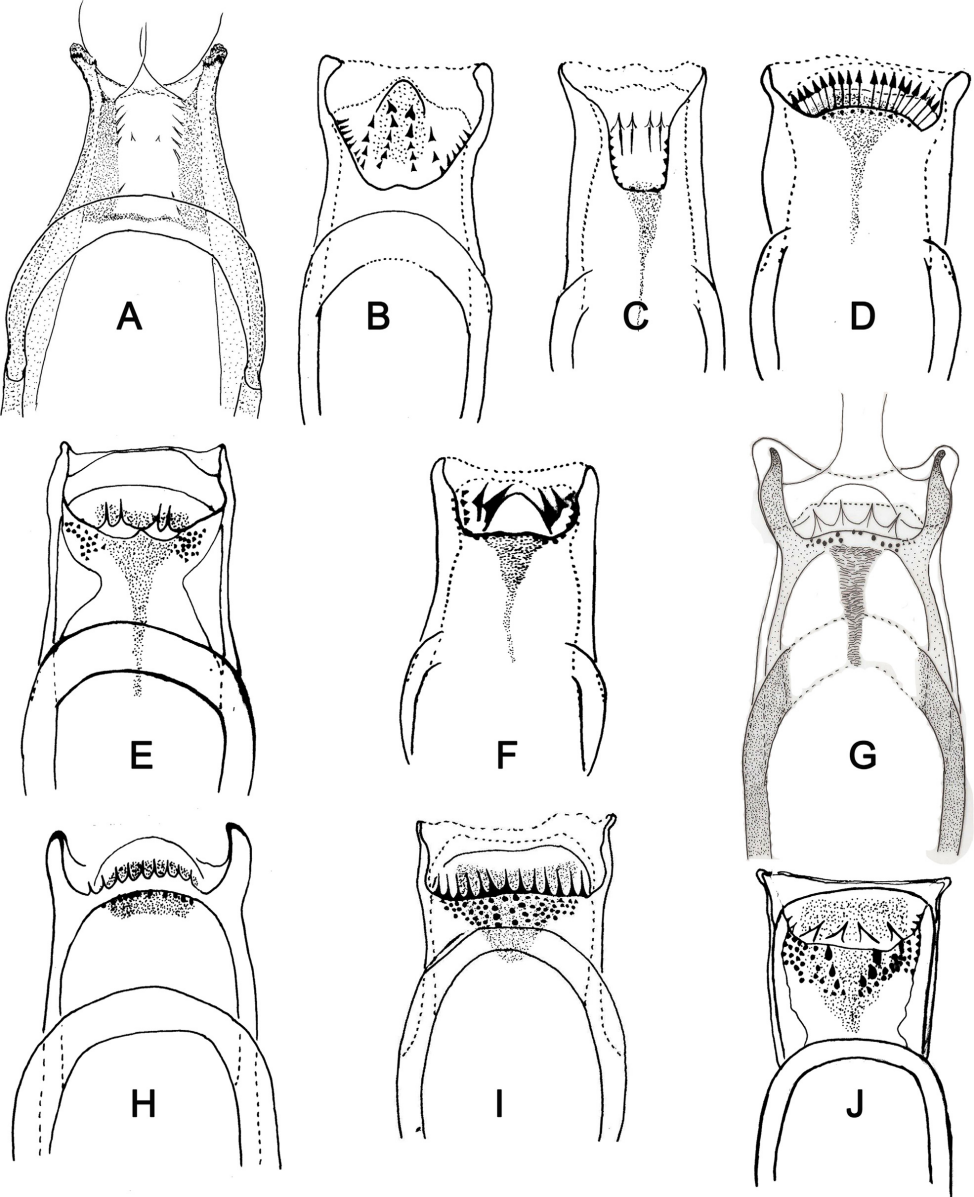
Cibarium of phlebotomine females. (A) *Edentomyia piauiensis*; (B) *Brumptomyia* sp; (C) *Micropygomyia pilosa*; (D) *Micropygomyia cayennensis*; (E) *Evandromyia walkeri*; (F) *Sciopemyia sordellii*; (G) *Lutzomyia* (*Helcocyrtomyia*) *kirigetiensis*; (H) *Lutzomyia longipalpis*; (I) *Trichophoromyia auraensis*; (J) *Psathyromyia lutziana*.

**Figure 9.**
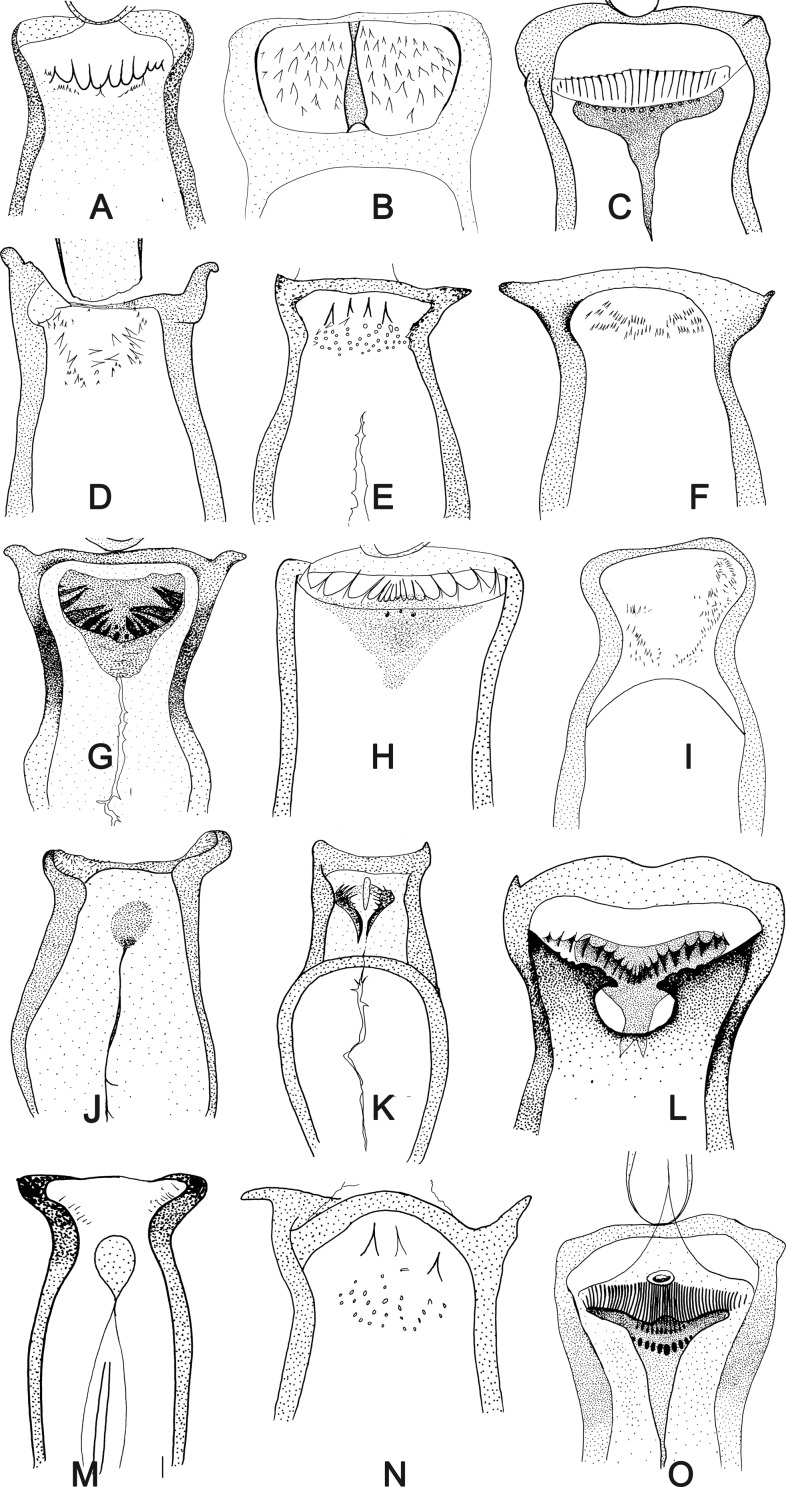
Cibarium of phlebotomine females. (A) *Australophlebotomus notteghemae*; (B) *Idiophlebotomus padillarum*; (C) *Sergentomyia* (*Vattieromyia*) *sclerosiphon*; (D) *Phlebotomus* (*Euphlebotomus*) *mascomai*; (E) *Phlebotomus* (*Madaphlebotomus*) *vaomalalae*; (F) *Sergentomyia bailyi*; (G) *Sergentomyia* (*Sergentomyia*) *phadangensis*; (H) *Sergentomyia hivernus*; (I) *Phlebotomus* (*Transphlebotomus*) *anatolicus*; (J) *Chinius eunicegalatiae*; (K) *Chinius samarensis*; (L) *Sergentomyia* (*Parrotomyia*) *babu*; (M) *Parvidens heishi*; (N) *Phlebotomus* (*Madaphlebotomus*) *vincenti*; (O) *Sergentomyia* (*Vattieromyia*) *namo.*

**Figure 10.**
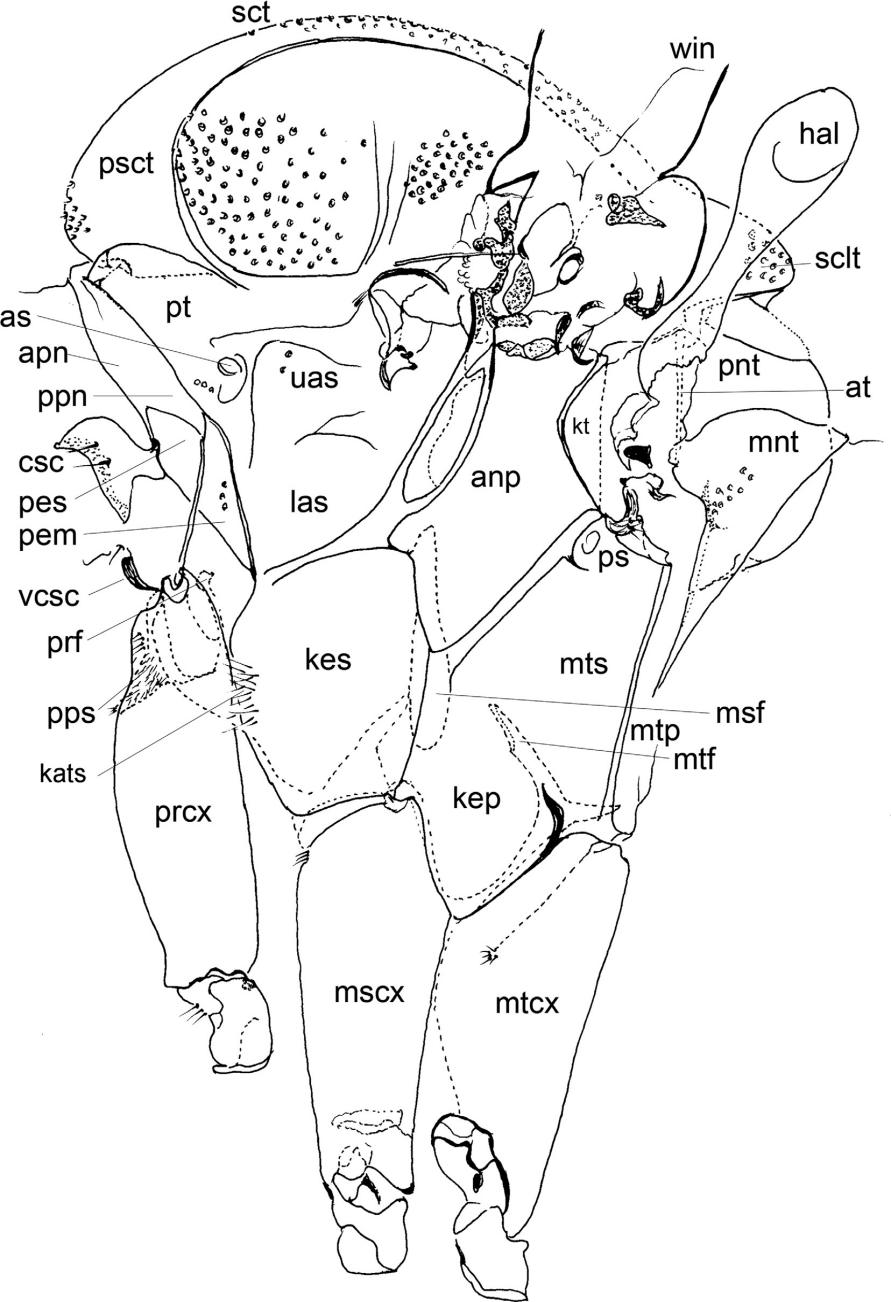
Sclerites of cervix and thorax of Phlebotominae. anp – anepimeron; apn – antepronotum; as – anterior spiracle; at – anatergite; csc – cervical sclerite with a pair of sensilla; hal – halter; kep – katepimeron; kes – katepisternum; kt – katatergitum; las – lower anepisternum; mnt – metanotum; mscx – mesocoxa; msf – mesofurca; mtcx – metacoxa; mtf – metafurca; mtp – metepimeron; mts – metepisternum; pem – proepimeron; pes – proepisternum; pnt – postnotum; ppn – postpronotum; pps – protuberance of the prosternum; prcx – procoxa; prf – profurca; ps – posterior spiracle; psct – prescutum; pt – paratergite; sclt – scutellum; sct – scutum; uas – upper anepisternum; vcsc – ventrocervical sclerite; win – wing. *Deanemyia samueli*.

**Figure 11.**
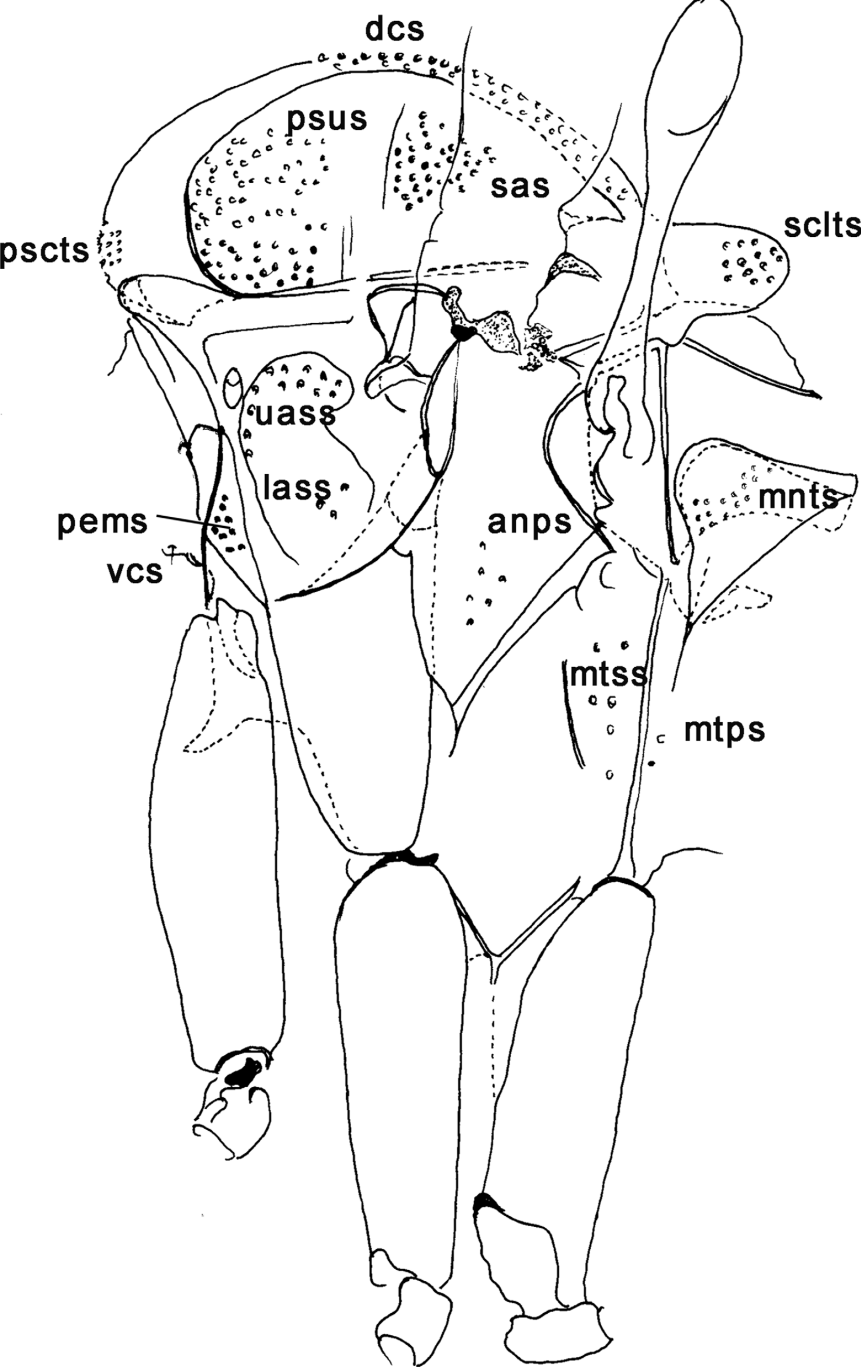
Setae on the thorax of phlebotomines: anps – anepimeral setae; dcs – dorsocentral setae; lass – lower anepisternal setae; mnts – metanotal setae; mtps – metepimeral setae; mtss – metepsiternal setae; pems – proepimeral setae; pscts – prescutal setae; psus – postsutural setae; sas – supralar setae; sctls – scutelar setae; uass – upper anepisternal setae; vcs – ventrocervical sensilla. *Brumptomyia pintoi*.

**Figure 12.**
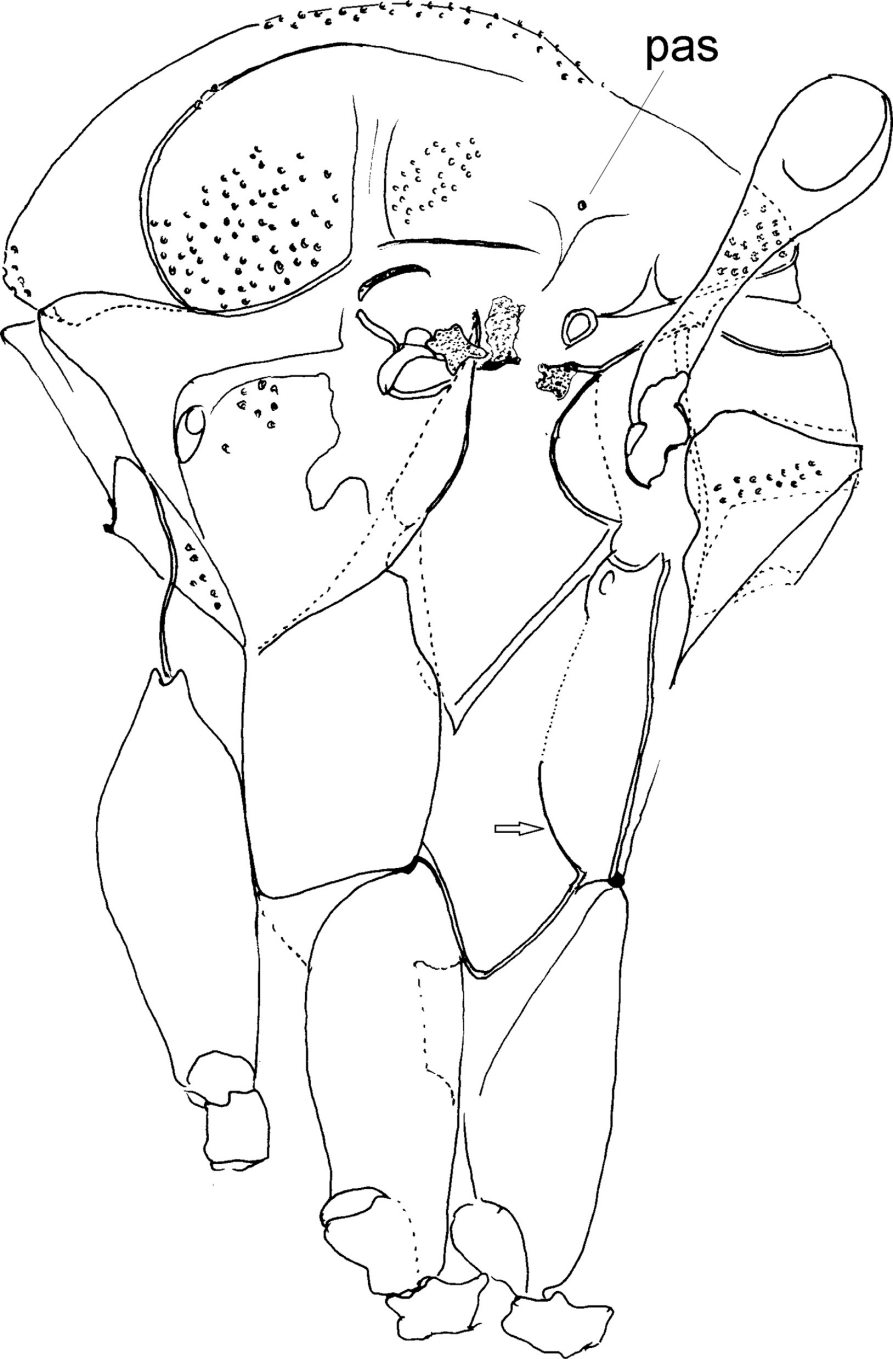
Thorax in lateral view of Phlebotominae with indication of characters in plesiomorphic state: arrow – indicating the long suture separating the katepimeron and metepisternum; pas – postalar seta. *Oligodontomyia toroensis*.

**Figure 13.**
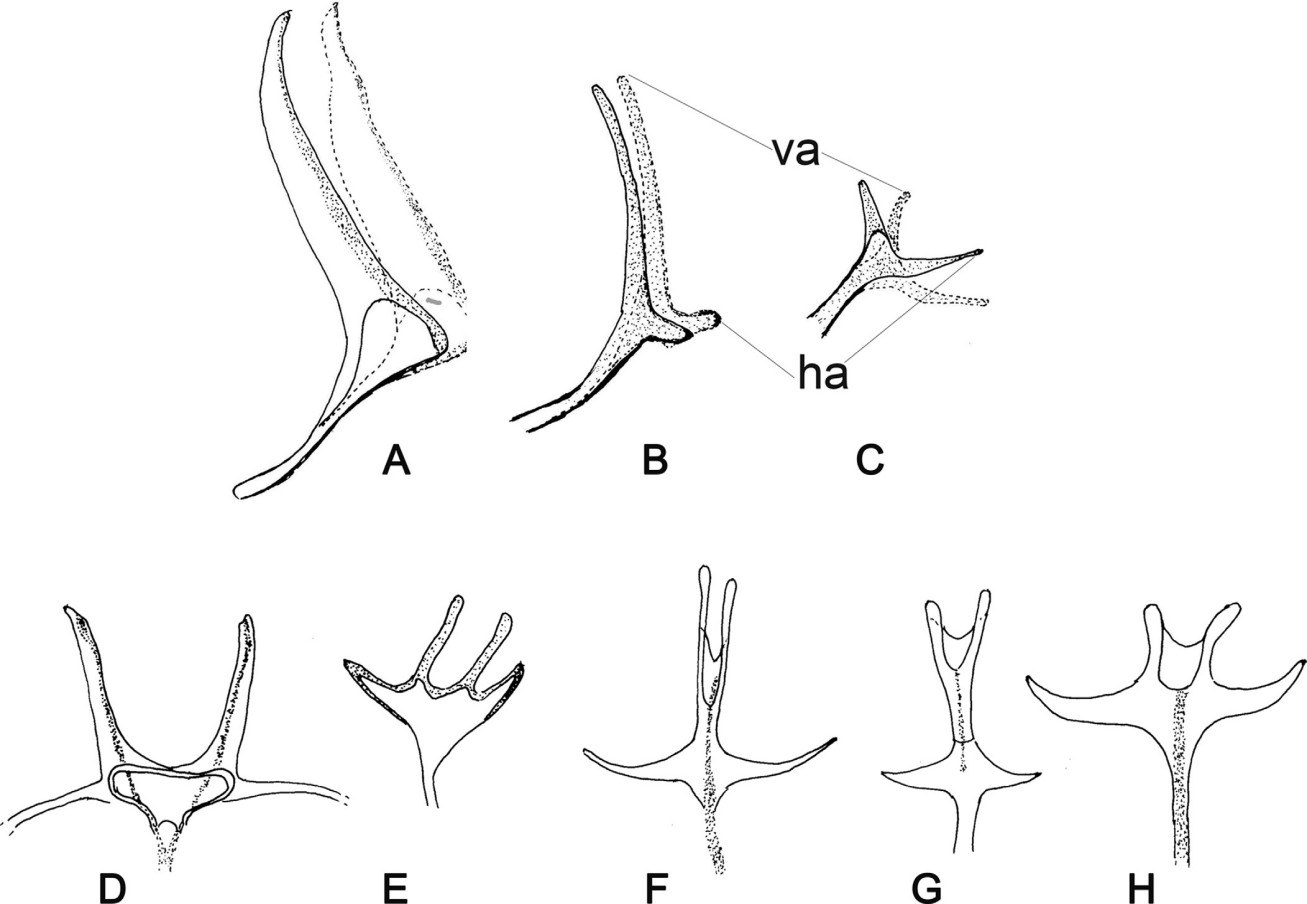
Metafurca of Phlebotominae: (A–C) Lateral view: (A) completely separate vertical arms and atrophied horizontal arms: *Warileya phlebotomanica*; (B) completely separate vertical arms and short horizontal arms: *Warileya nigrosaculla*; (C) completely separate vertical arms and long horizontal arms: *Chinius samarensis*. (D–H) Frontal view: (D) completely separate vertical arms and atrophied horizontal arms: *Wa. phlebotomanica*; (E) completely separate vertical and short horizontal arms: *Chinius eunicegalatiae*; (F) united long vertical and horizontal arms: *Sergentomyia minuta*; (G) united long vertical and short horizontal arms: *Bichromomyia flaviscutellata*; (H) united short vertical and long horizontal arms: *Brumptomyia brumpti*.

**Figure 14.**
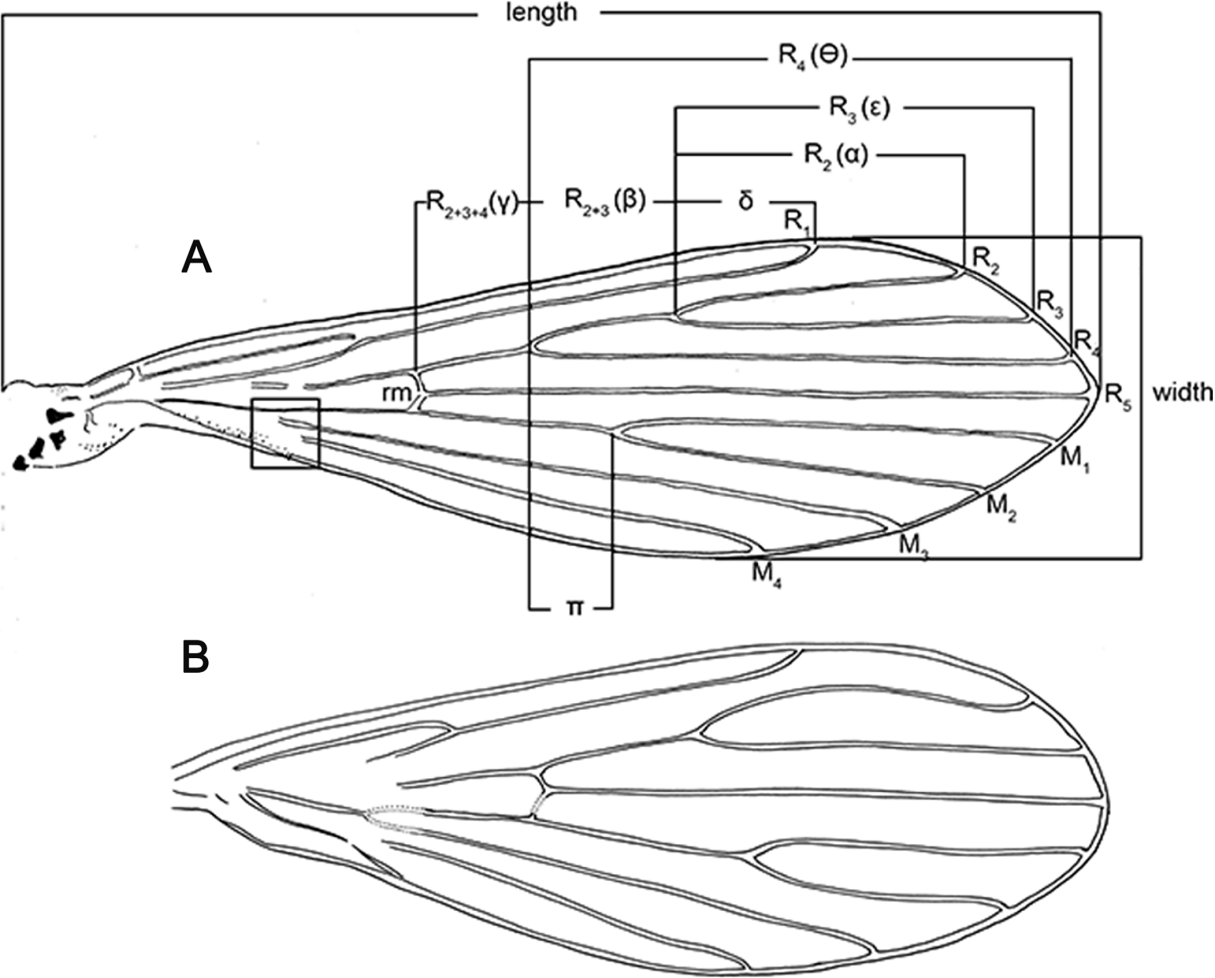
Wing of Phlebotominae. (A) Main indices; (B) wing with of fusion of R2 and R3: *Chinius eunicegalatiae.*

**Figure 15.**
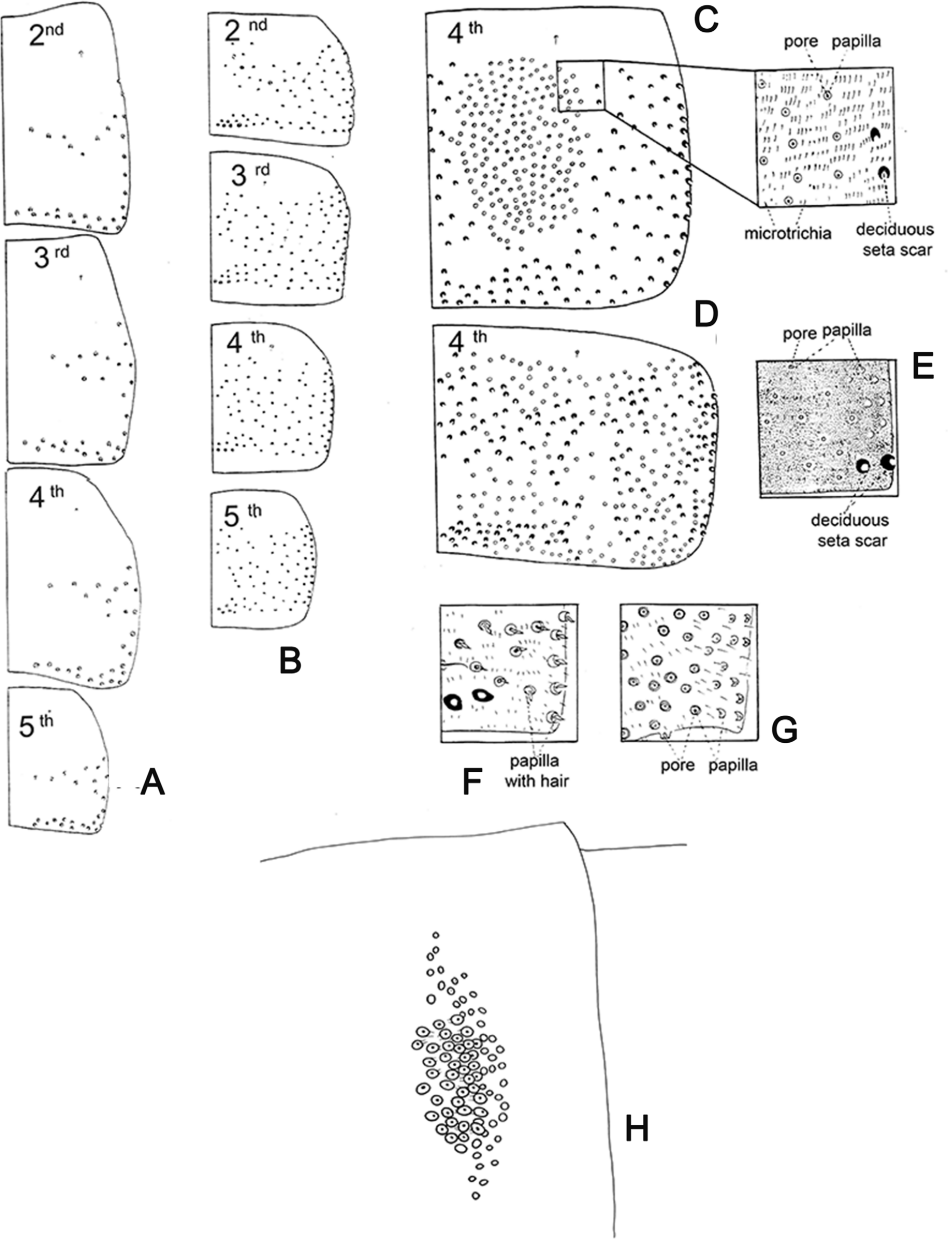
Abdominal tergites of Phlebotominae, showing arrangements of the deciduous bristles and tergal papillae and aspects of the tergal papillae and “trumpets glands”. (A, B) 2nd–5th male tergites with the arrangement of the deciduous bristles. (A) Two transverse bands: *Warileya phlebotomanica*. (B) Randomly: *Nyssomyia intermedia*. (C, D) Distribution and aspects of the tergal papillae on 4th tergite; (C) restricted to the central area: *Lutzomyia longipalpis*; (D) dispersed over the surface of the tergite, among the deciduous bristle scars: *Pintomyia fischeri*. (E–G) Aspects of the papillae on the 6th tergite: (E) papillae without hair and without clear demarcation of their borders; (F) papillae with hair: *Brumptomyia cardosoi*; (G) papillae without hair and with clear demarcation of their borders: *Evandromyia walkeri*. (H) “Trumpet glands” of the fourth abdominal tergite: *Chinius samarensis*.

**Figure 16.**
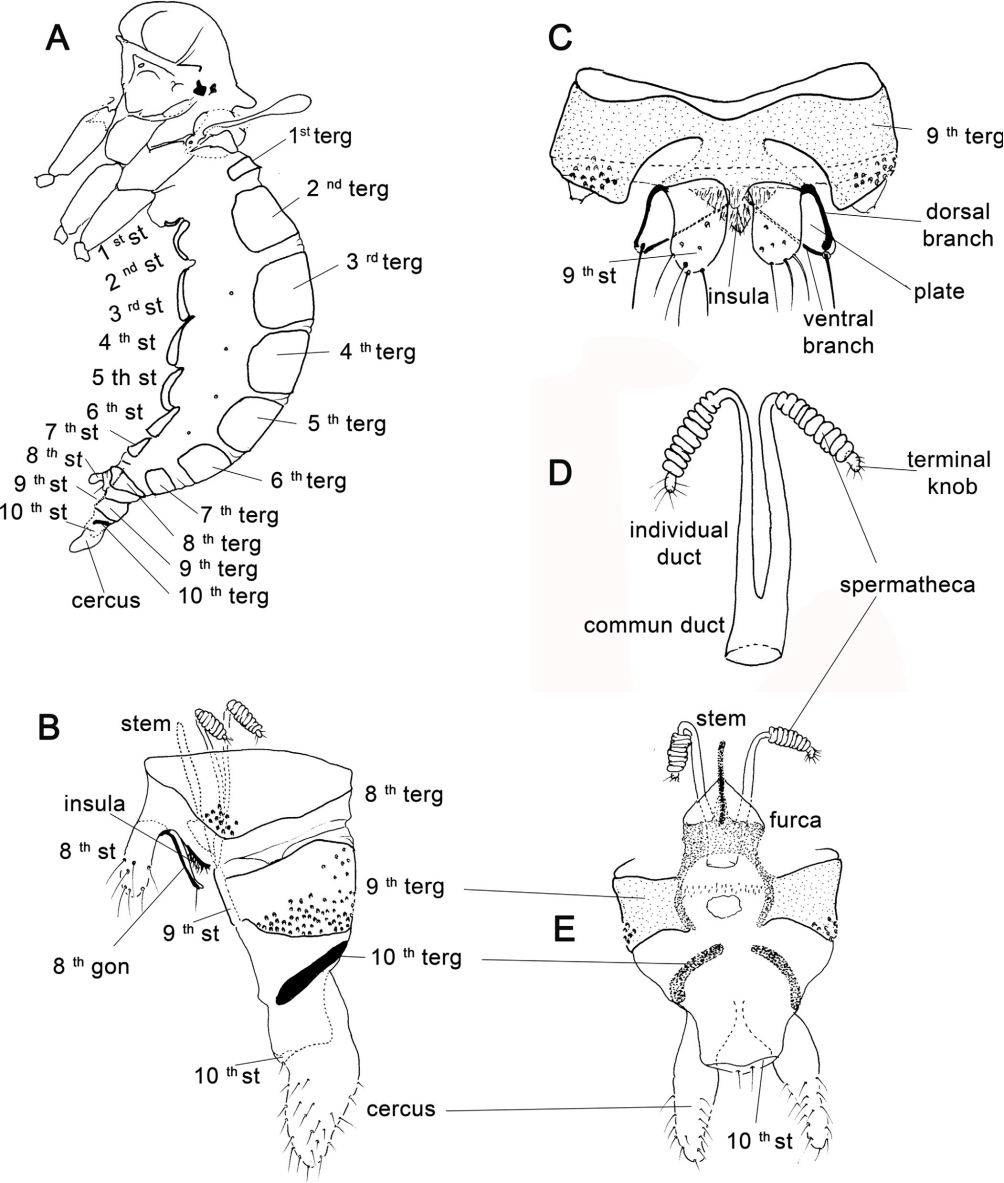
Abdomen and genitalia of Phlebotomine female. (A) abdomen in lateral view; (B) genitalia in lateral view; (C) 8th segment in ventral view; (D) 9th segment, 10th segment and cerci in ventral view. *Nyssomyia neivai*. gon – gonopod; st – sternite; stem – fork stem; terg – tergite.

**Figure 17.**
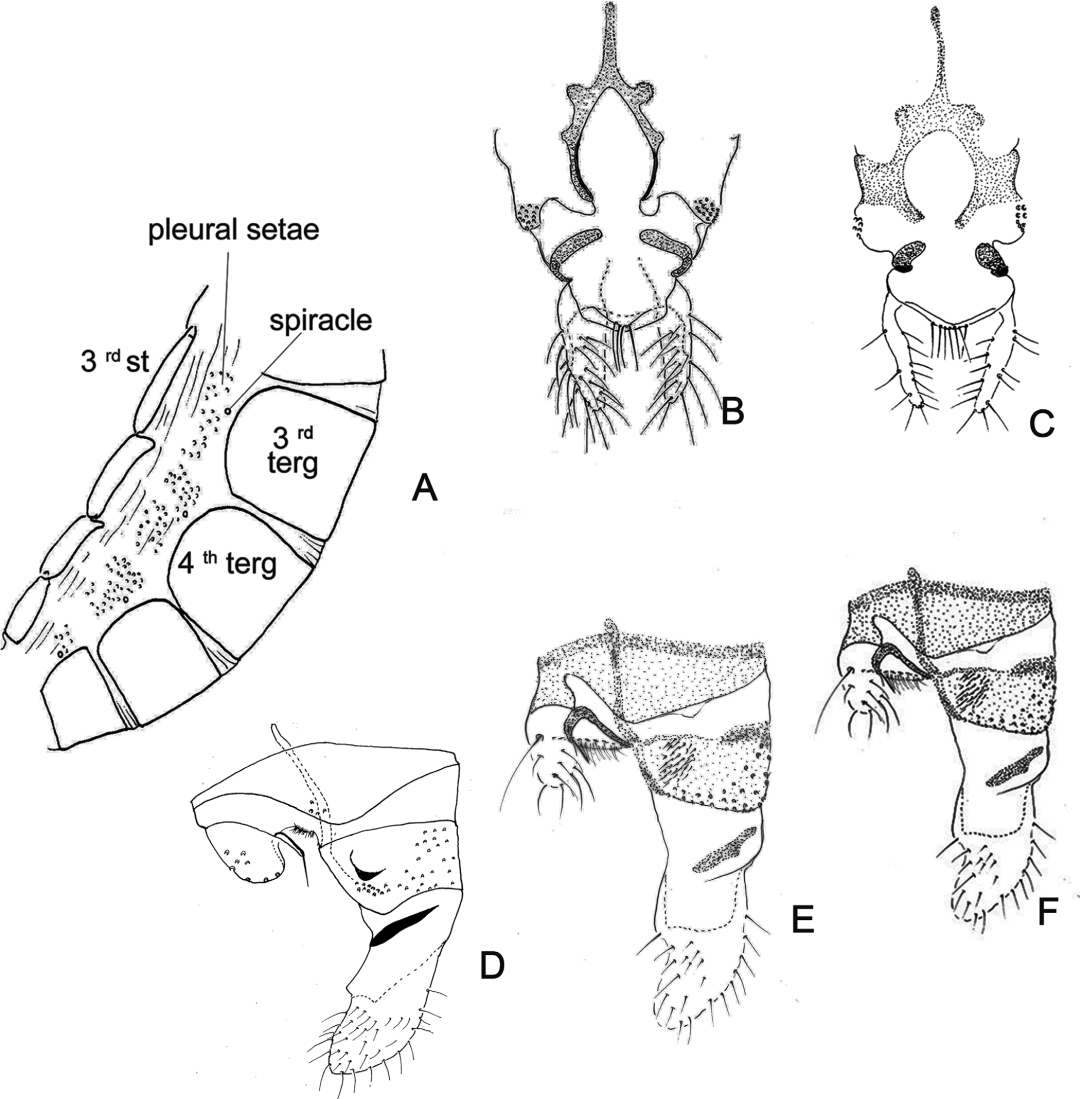
Some structures of the abdomen and the genitalia of phlebotomine females. (A) 3rd–6th abdominal segments showing the pleural setae: *Lutzomyia* (*Tricholateralis*) *sherlocki.* (B, C) 10th sternite showing non-deciduous setae in the median region: (B) *Micropygomyia vexator*, (C) *Sergentomyia minuta*. (D) 9th segment showing a sclerotized protuberance on the tergite: *Migonemyia* (*Migonemyia*) *rabelloi*, (E) spicules in 9th and 10th tergites: *Lutzomyia* (*Tricholateralis*) *cruciata.* (F) 9th tergite with short bristles: *Sciopemyia sordellii*.

**Figure 18.**
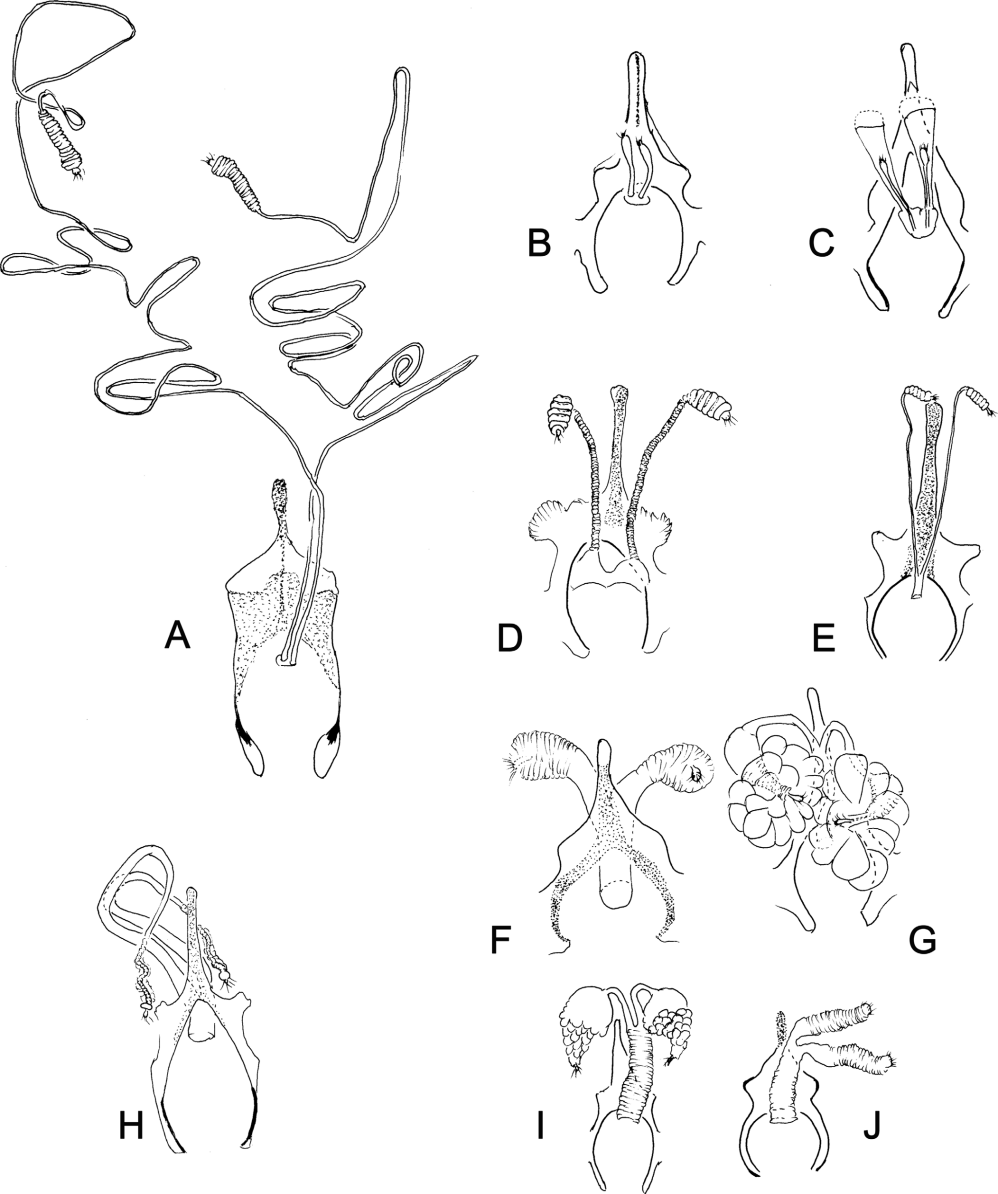
Spermathecae and genital fork aspects of Phlebotominae. (A) *Brumptomyia brumpti*; (B) *Migonemyia* (*Blancasmyia*) *gorbitzi*; (C) *Viannamyia tuberculata*; (D) *Phlebotomus* (*Phlebotomus*) *papatasi*; (E) *Lutzomyia* (*Lutzomyia*) *longipalpis*; (F) *Dampfomyia* (*Coromyia*) *vespertilionis*; (G) *Dampfomyia* (*Dampfomyia*) *anthophora*; (H) *Micropygomyia* (*Coquillettimyia*) *vexator*; (I) *Evandromyia* (*Evandromyia*) *saulensis*; (J) *Ev.* (*Eva.) infraspinosa*.

**Figure 19.**
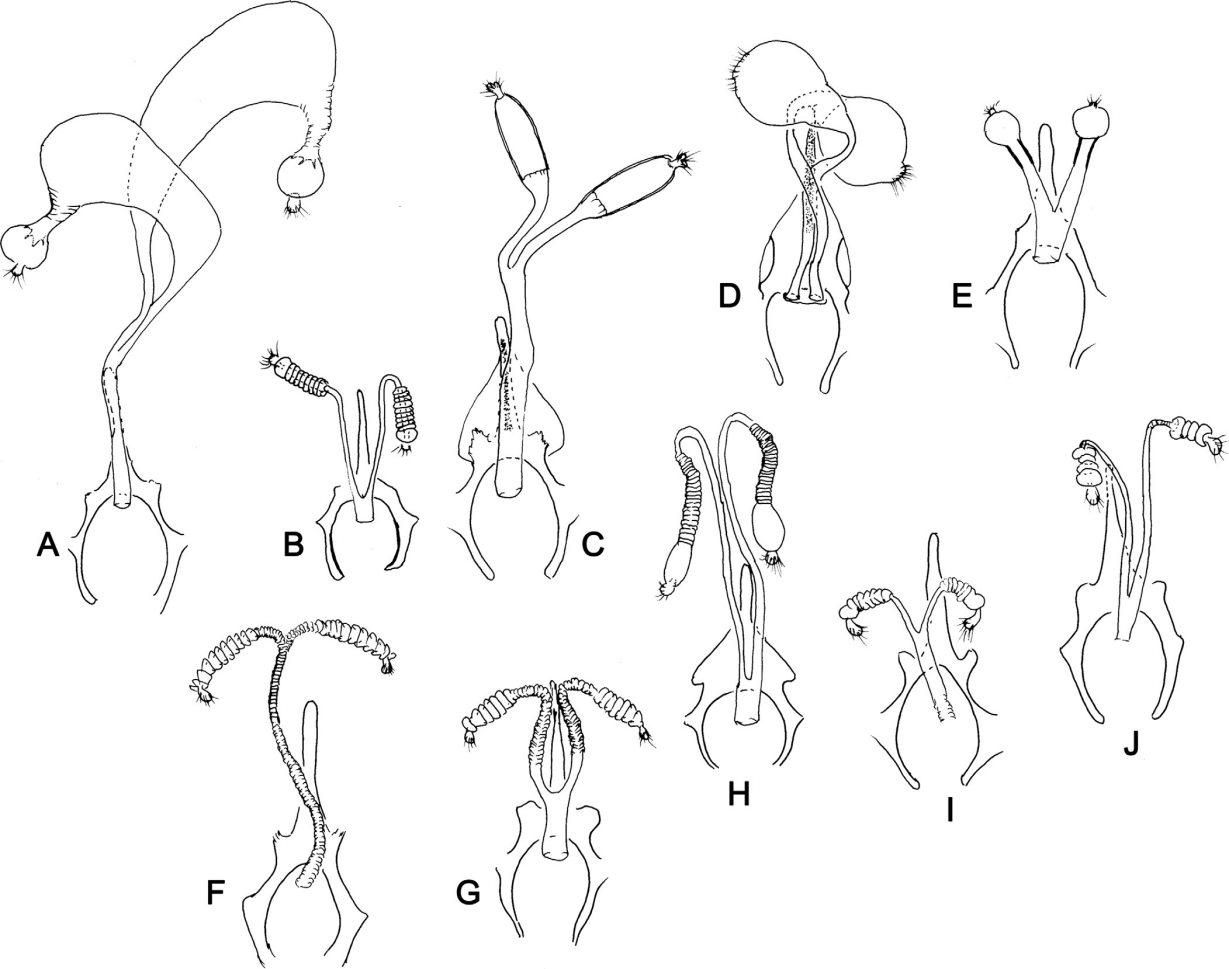
Spermathecae and genital fork aspects of Phlebotominae. (A) *Evandromyia* (*Barrettomyia*) *tupynambai*; (B) *Psathyromyia* (*Psathyromyia*) *lanei*; (C) *Pa.* (*Psa.) shannoni*; (D) *Pa.* (*Forattiniella*) *aragaoi*; (E) *Pa.* (*For.) lutziana*; (F) *Psychodopygus panamensis*; (G) *Ps. chagasi*; (H) *Trichophoromyia auraensis*; (I) *Bichromomyia flaviscutellata*; (J) *Martinsmyia alphabetica*.

**Figure 20.**
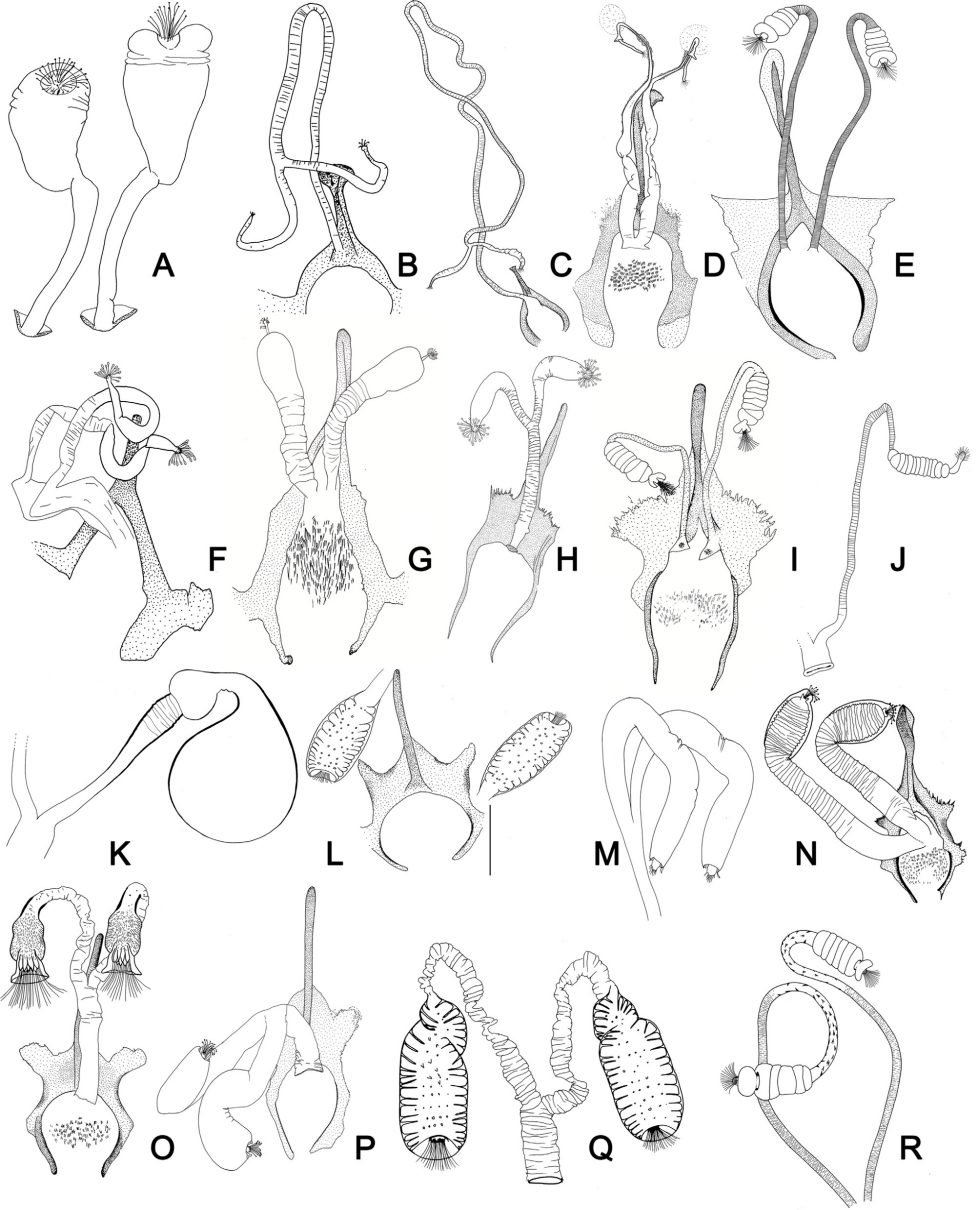
Spermathecae and genital fork aspects of Phlebotominae. (A) *Australophlebotomus notteghemae*; (B) *Chinius eunicegalatiae*; (C) *Chinius samarensis*; (D) *Phlebotomus* (*Madaphlebotomus*) *vincenti*; (E) *Ph.* (*Paraphlebotomus*) *sergenti*; (F) *Idiophlebotomus padillarum*; (G) *Parvidens heishi*; (H) *Ph.* (*Euphlebotomus*) *barguesae*; (I) *Ph.* (*Par.*) *chabaudi*; (J) *Phlebotomus* (*Larroussius*) *major*; (K) *Spelaeomyia moucheti*; (L) *Sergentomyia* (*Rondanomyia*) *goodmani comorensis*; (M) *Se. hivernus*; (N) *Ph.* (*Transphlebotomus*) *economidesi*; (O) *Se.* (*Vattieromyia*) *namo*; (P) *Se.* (*Sergentomyia*) *phadangensis*; (Q) *Sergentomyia* (*Ron.*) *goodmani*; (R) *Ph.* (*Par.*) *mireillae.*

**Figure 21.**
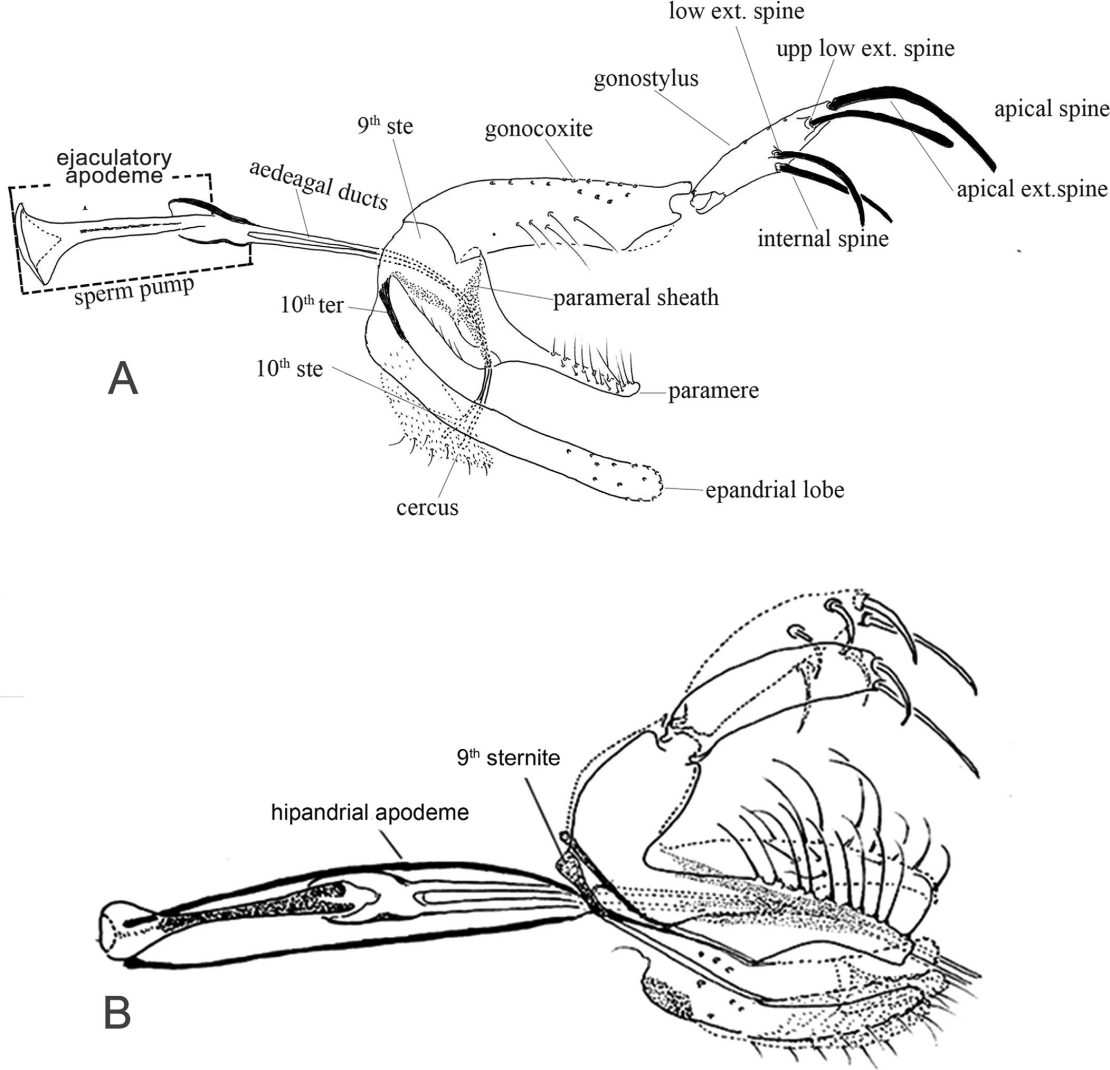
Lateral view of male genitalia of Phlebotominae. (A) *Nyssomyia neivai*; (B) *Warileya nigrosaccula*.

Many pertinent characters of Phlebotominae that are currently used for their identification and classification have been described by authors using distinct terminology. This practice has been criticized by other dipterists because, in addition to hindering studies of homology within Phlebotominae, it also makes comparisons among supra-specific taxa difficult. This is particularly problematic for characters of the male terminalia. To this end, we suggest that terminology used in phlebotomine taxonomy be more closely aligned with that used in general dipterology [2, 7]. In order to standardize the descriptions and re-descriptions of phlebotomine species, a list of characters (all body parts and their appendages and sensorial structures, such as spines, setae, papillae, sensilla, sutures, etc.) deserving of comment and illustration is proposed (Table 1). Furthermore, in order to contribute to the standardization of terminology for structures that have been frequently used in phlebotomine taxonomic studies, a list is provided (Table 2) including suggested terms in English and synonyms of these terms that have been used in the past by various authors, in French and Portuguese. Some characters have been infrequently used in published descriptions and may thus be poorly known; for this reason and for the purpose of illustrating the morphological diversity of some characters, drawings have been provided with the corresponding terminology in each legend (Figures 1–28). A drawing is preferable to a photo, but sometimes for structures such as the pharyngeal armature, a photo can supplement or replace a drawing. We posit that the list of characters suggested permits a detailed description of the species and provides elements for species distinction and phylogenetic studies.

**Figure 22.**
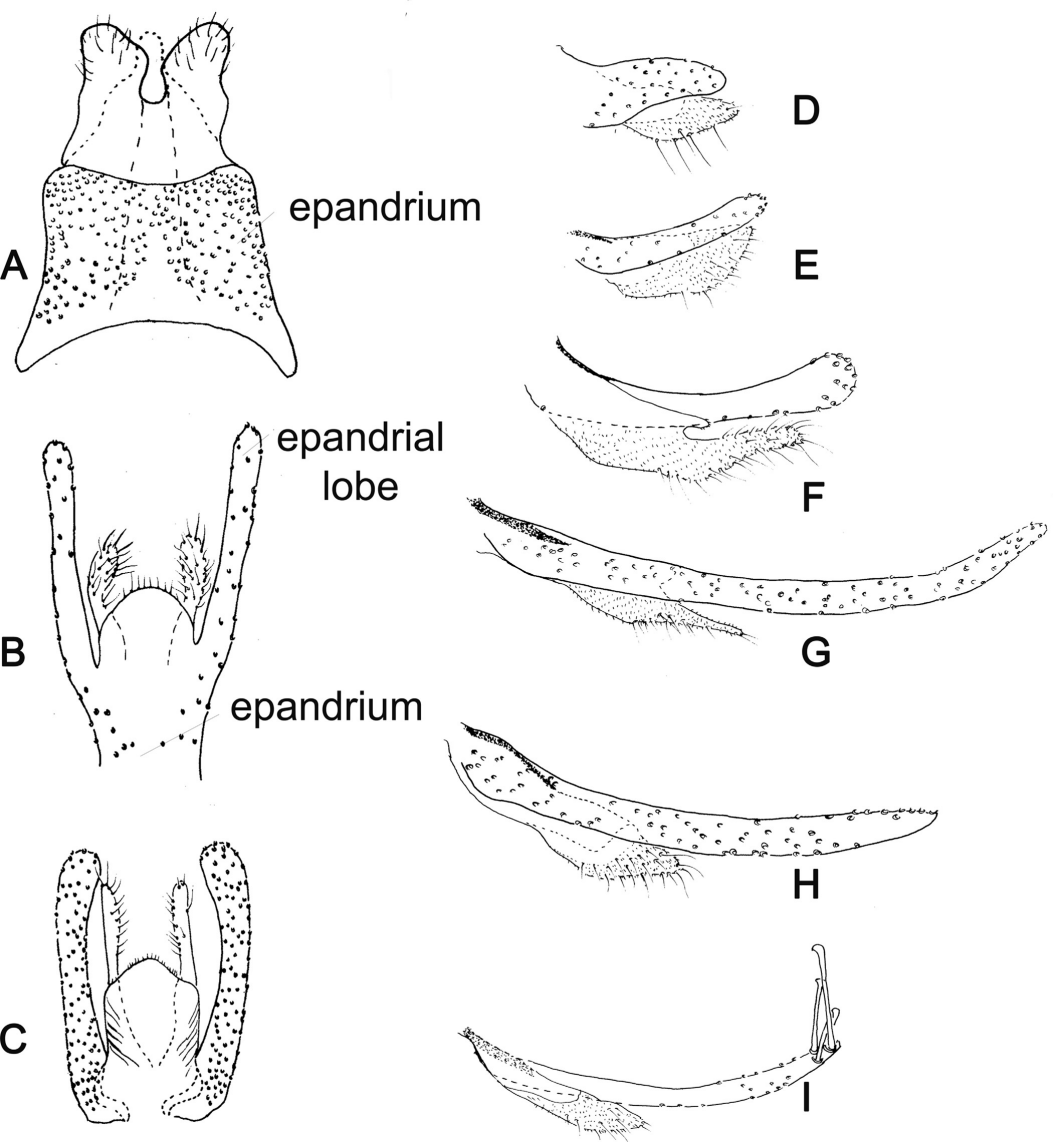
(A–C) Ventral view of the epandrium and of epandrial lobes and cerci of Phlebotominae. (A) Bruchomyiinae (*Bruchomyia* sp); (B, C) Phlebotominae: (B) *Warileya nigrosaccula*; (C) *Psychodopygus chagasi*. (D–I) Lateral view of epandrial lobe and cercus of Phlebotominae: (D) *Hertigia hertigi*; (E) *Sciopemyia sordellii*; (F) *Psychodopygus chagasi*; (G) *Trichopygomyia longispina*; (H) *Evandromyia* (*Aldamyia*) *walkeri*; (I) *Evandromyia* (*Evandromyia*) *infraspinosa.*

**Figure 23.**
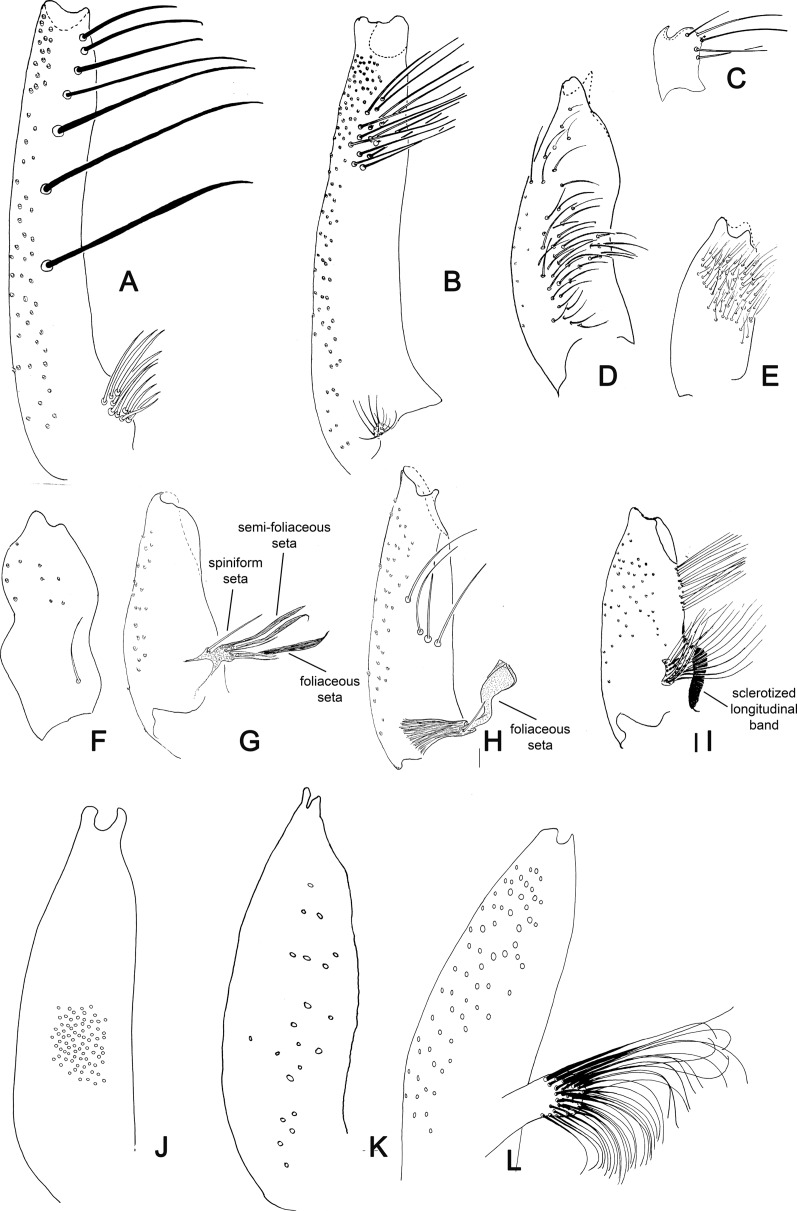
(A–C) Lateral view of gonocoxites of Phlebotominae. (A) *Brumptomyia brumpti*; (B) *Phlebotomus papatasi*; (C) *Migonemyia* (*Blancasmyia*) *gorbitzi*; (D) *Trichophoromyia auraensis*; (E) *Micropygomyia* (*Micropygomyia*) *pilosa*; (F) *Psychodopygus chagasi*; (G) *Lutzomyia* (*Tricholateralis*) *carvalhoi*; (H) *Lutzomyia* (*Lutzomyia*) *almerioi*; (I) *Pintomyia* (*Pifanomyia*) *verrucarum*; (J) *Phlebotomus hindustanicus*; (K) *Sergentomyia dentata*; (L) *Phlebotomus mireillae.*

**Figure 24.**
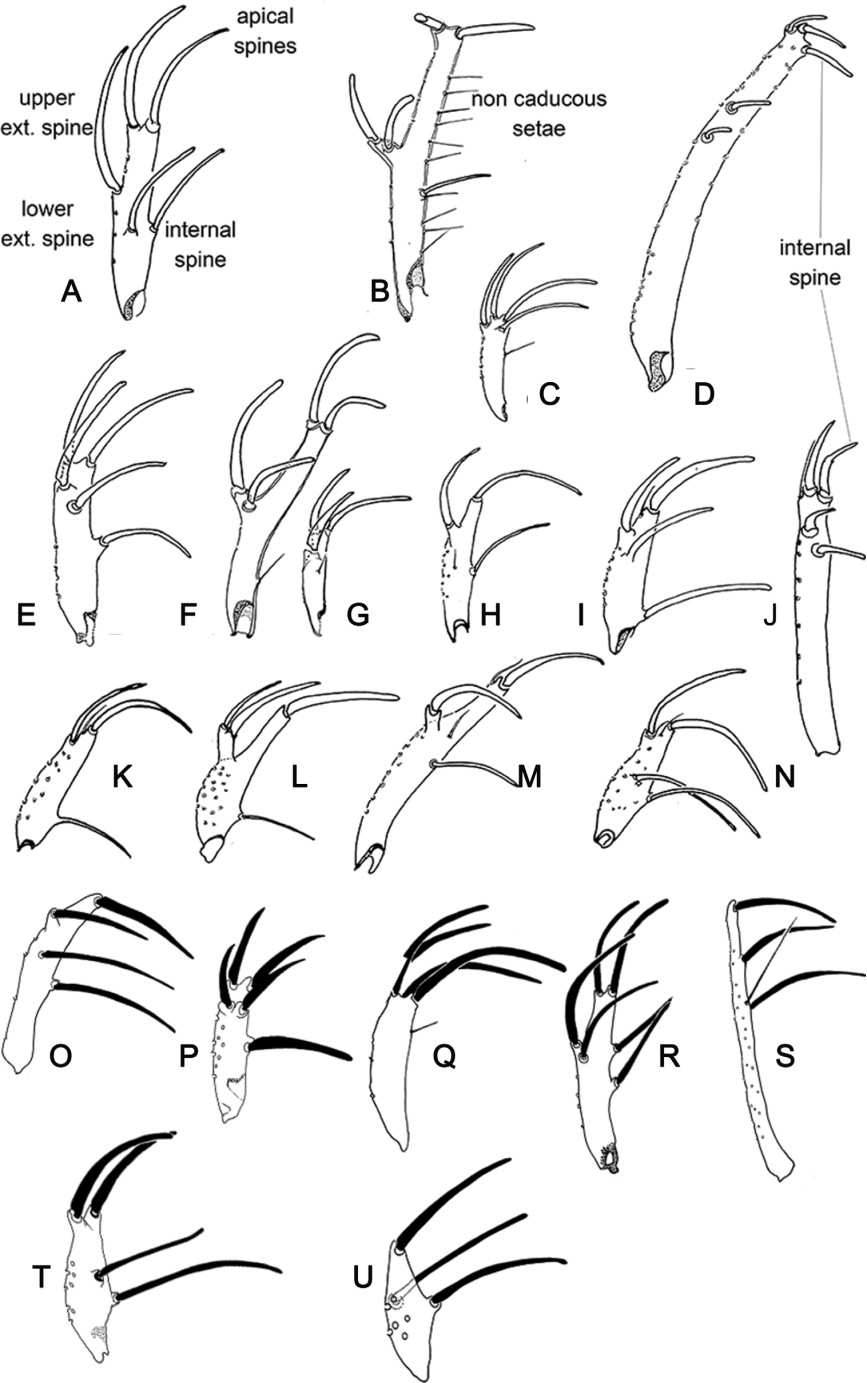
Lateral view of gonostyles of Phlebotominae. (A) *Micropygomyia chiapanensis*; (B) *Brumptomyia cardosoi*; (C) *Sergentomyia minuta*; (D) *Phlebotomus papatasi*; (E) *Edentomyia piauiensis*; (F) *Oligodontomyia toroensis*; (G) *Deanemyia samueli*; (H) *Micropygomyia pilosa*; (I) *Pintomyia* (*Pifanomyia*) *sauroida*; (J) *Migonemyia* (*Blancasmyia*) *gorbitzi*; (K) *Pintomyia* (*Pifanomyia*) *serrana*; (L) *Evandromyia* (*Evandromyia*) *correalimai*; (M) *Pressatia triacantha*; (N) – *Evandromyia saulensis*; (O) *Chinius samarensis*; (P) *Parvidens heishi*; (Q) *Sergentomyia – Sergentomyia* (*Sergentomyia*) *dentate*; (R) *Phlebotomus* (*Transphlebotomus*) *economidesi*; (S) *Idiophlebotomus padillarum*; (T) *Phlebotomus* (*Paraphlebotomus*) *andrejevi*; (U) *Phlebotomus* (*Legeromyia*) *multihamatus.*

**Figure 25.**
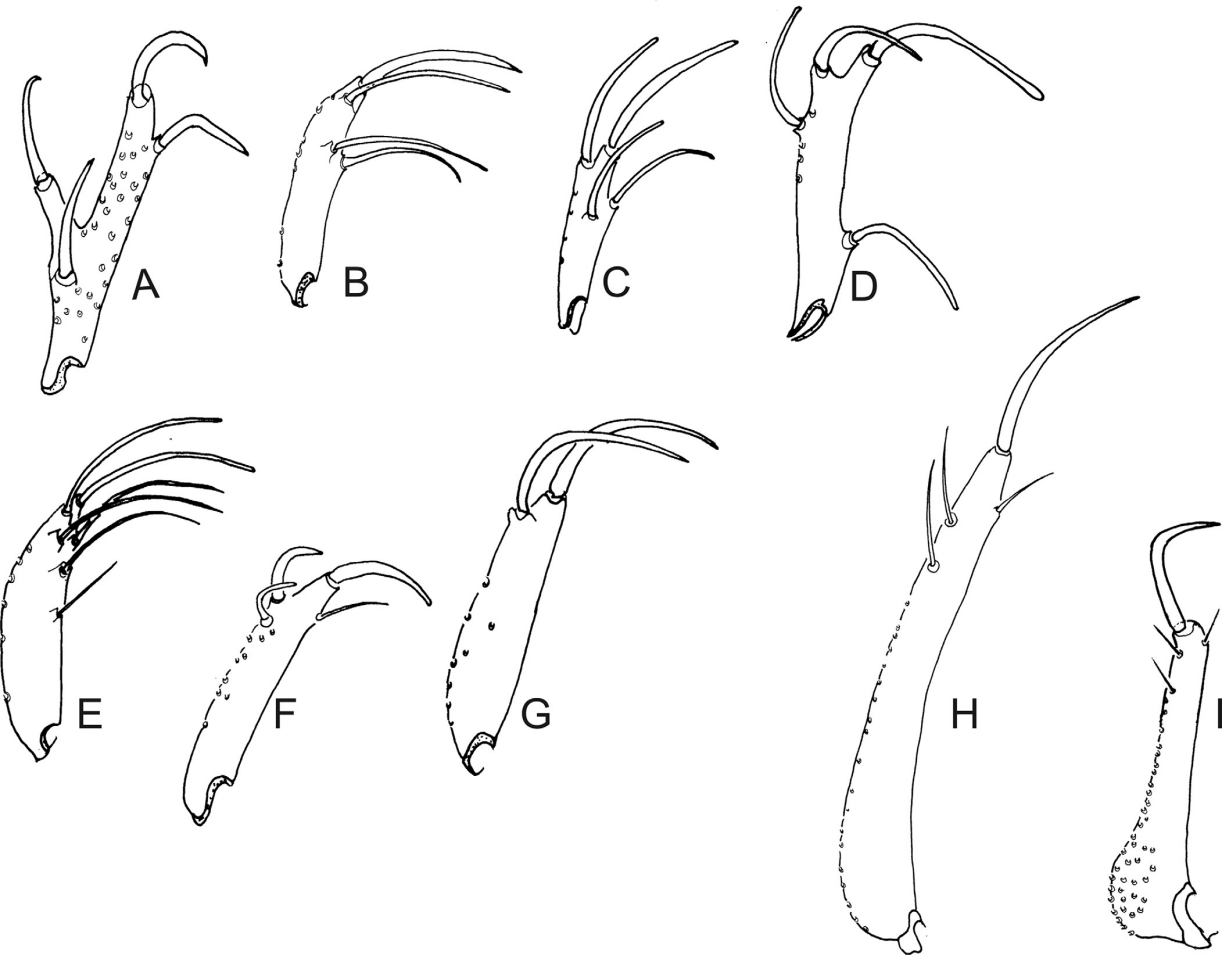
Lateral view of gonostyles of Phlebotominae. (A) *Viannamyia tuberculata*; (B) *Psathyromyia lanei*; (C) *Bichromomyia flaviscutellata*; (D) *Trichophoromyia auraensis*; (E) *Martinsmyia alphabetica*; (F) *Psychodopygus panamensis*; (G) *Psychodopygus bispinosus*; (H) *Psychodopygus geniculatus*; (I) *Psychodopygus chagasi.*

**Figure 26.**
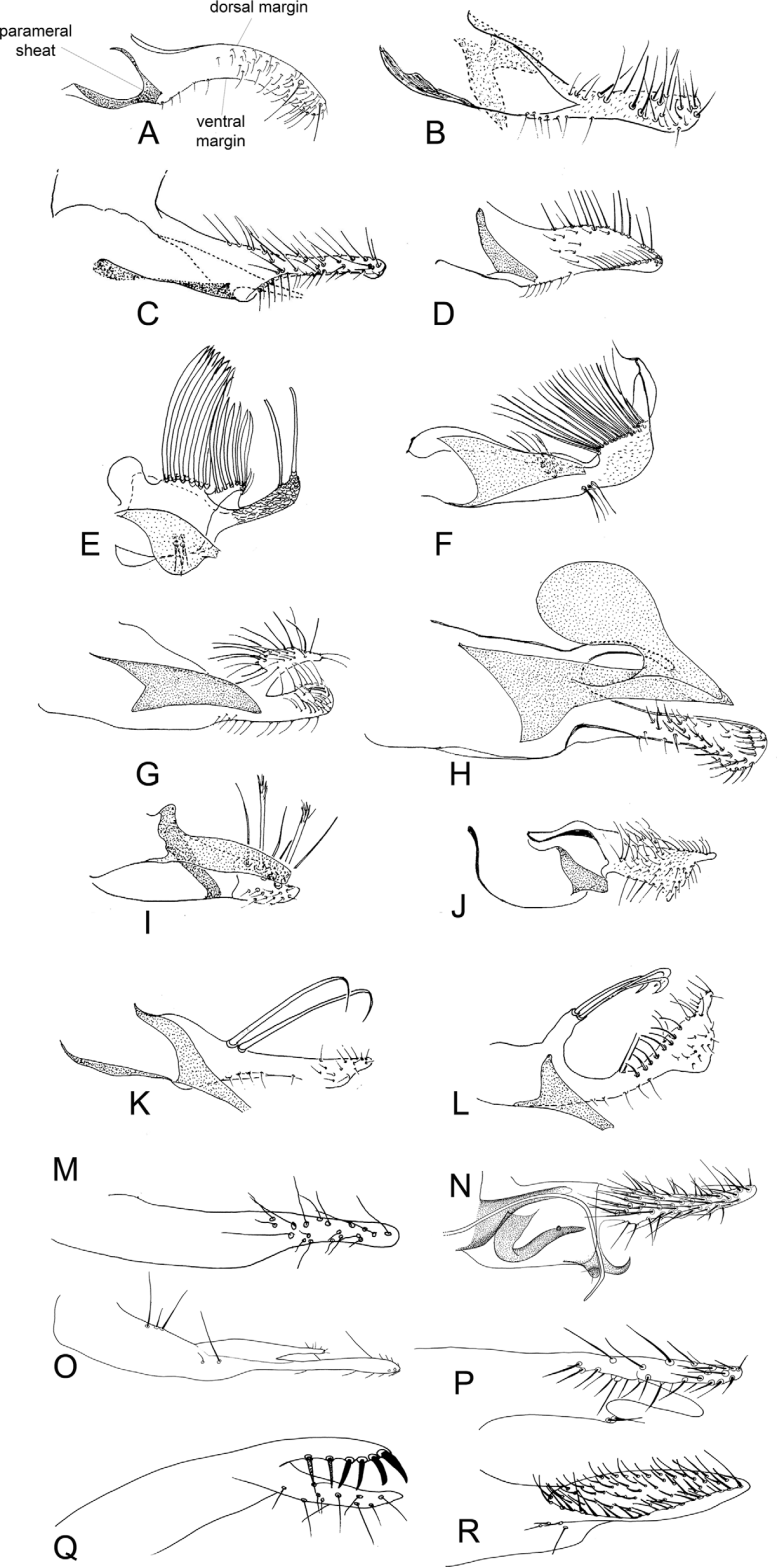
Lateral view of paramere and parameral sheath of Phlebotominae. (A) *Migonemyia* (*Blancasmyia*) *gorbitzi*; (B) *Evandromyia* (*Aldamyia*) *walkeri*; (C) *Lutzomyia* (*Helcocyrtomyia*) *guderiani*; (D) *Psathyromyia* (*Psathyromyia*) *lanei*; (E) *Psychodopygus panamensis*; (F) *Psychodopygus chagasi*; (G) *Trichopygomyia longispina*; (H) *Trichopygomyia dasypodogeton*; (I) *Viannamyia tuberculata*; (J) *Pressatia triacantha*;(K) *Lutzomyia* (*Lutzomyia*) *longipalpis*; (L) *Lutzomyia* (*Lutzomyia*) *dispar*; (M) *Sergentomyia* (*Sergentomyia*) *dentate*; (N) *Phlebotomus* (*Legeromyia*) *multihamatus*; (O) *Idiophlebotomus padillarum*; (P) *Phlebotomus* (*Euphlebotomus*) *barguesae*; (Q) *Parvidens heishi*; (R) *Phlebotomus* (*Paraphlebotomus*) *andrejevi*.

**Figure 27.**
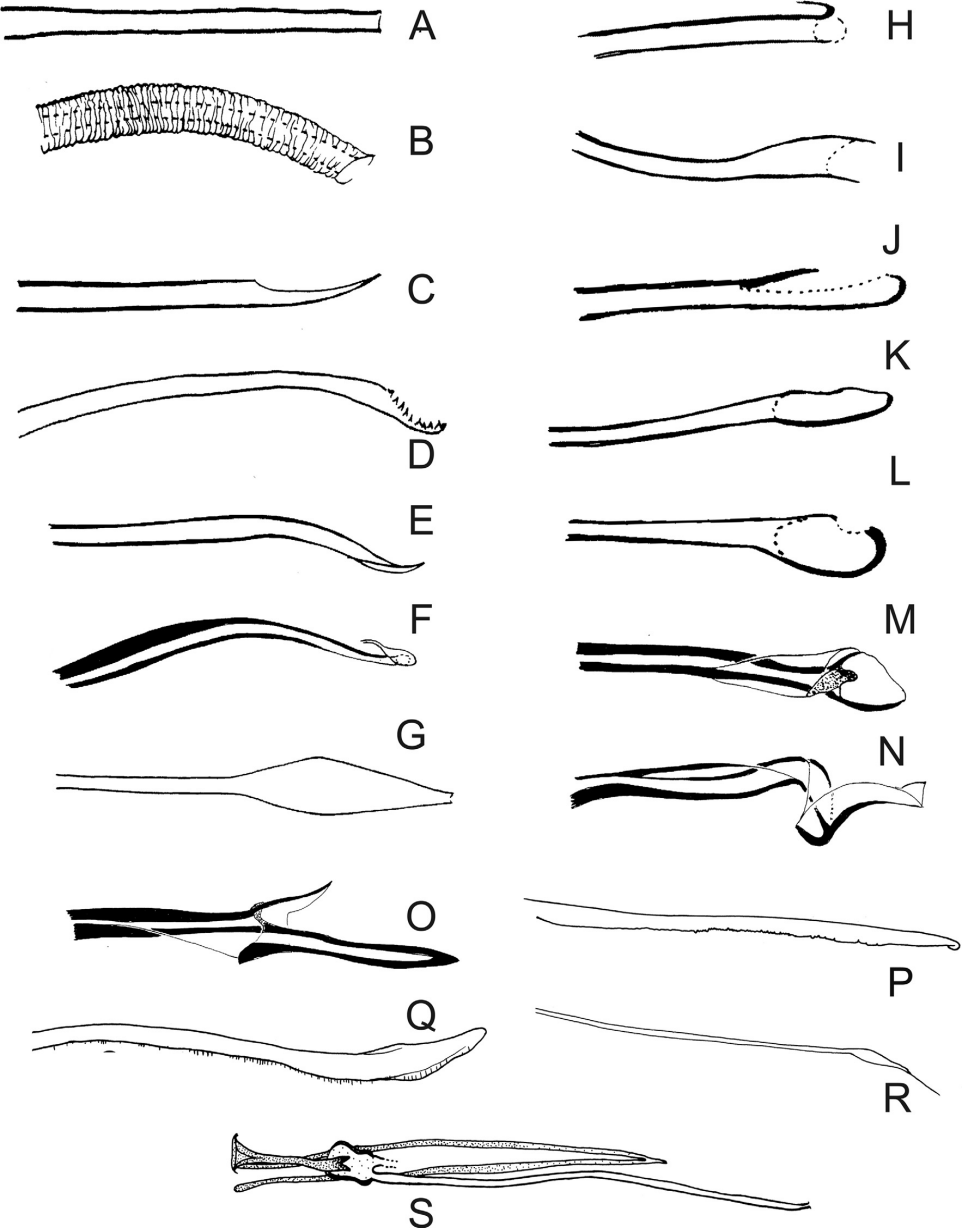
(A–R) Lateral view of terminal region of aedeagal ducts of Phlebotominae. (S) Genital pump, aedeagal ducts and hypandrial apodemes (abdominal rods). (A) Blunt apex: *Lu. longipalpis*; (B) striated duct with blunt apex: *Ev. brachyphalla*; (C) apex beveled: *Pa. shannoni*; (D) curved and toothed apex: *Ma. gasparviannai*; (E) curved and beveled apex: *Mi. longipennis*; (F) duct curved in its preapical region and apex provided with appendix: *Vi. tuberculata*; (G) lozenge apex: *Pa. runoides*; (H) apex with barbs: *Ny. yuilli pajoti*; (I–J) bifurcated apex: (I) *Ny. whitmani*; (J) *Nyssomyia anduzei*, (K) ladle-shaped apex: *Ny. intermedia*, (L) spoon shaped or knife to eat fish shaped: *Ny. neivai*; (M) clavate apex: *Ev. walkeri*; (N) duct with curved preapical region and blunt apex: *Pa. aragaoi*; (O) strongly sclerotized bifurcated apex: *Ev. lenti*; (P) irregular side: *Se. anka*; (Q) enlarged at the top: *Se. sclerosiphon*; (R) with apical inflated portion: *Id. padillarum*. (S) Terminal region of aedeagal ducts with blunt apex: *Id. nicolegerae*.

**Figure 28.**
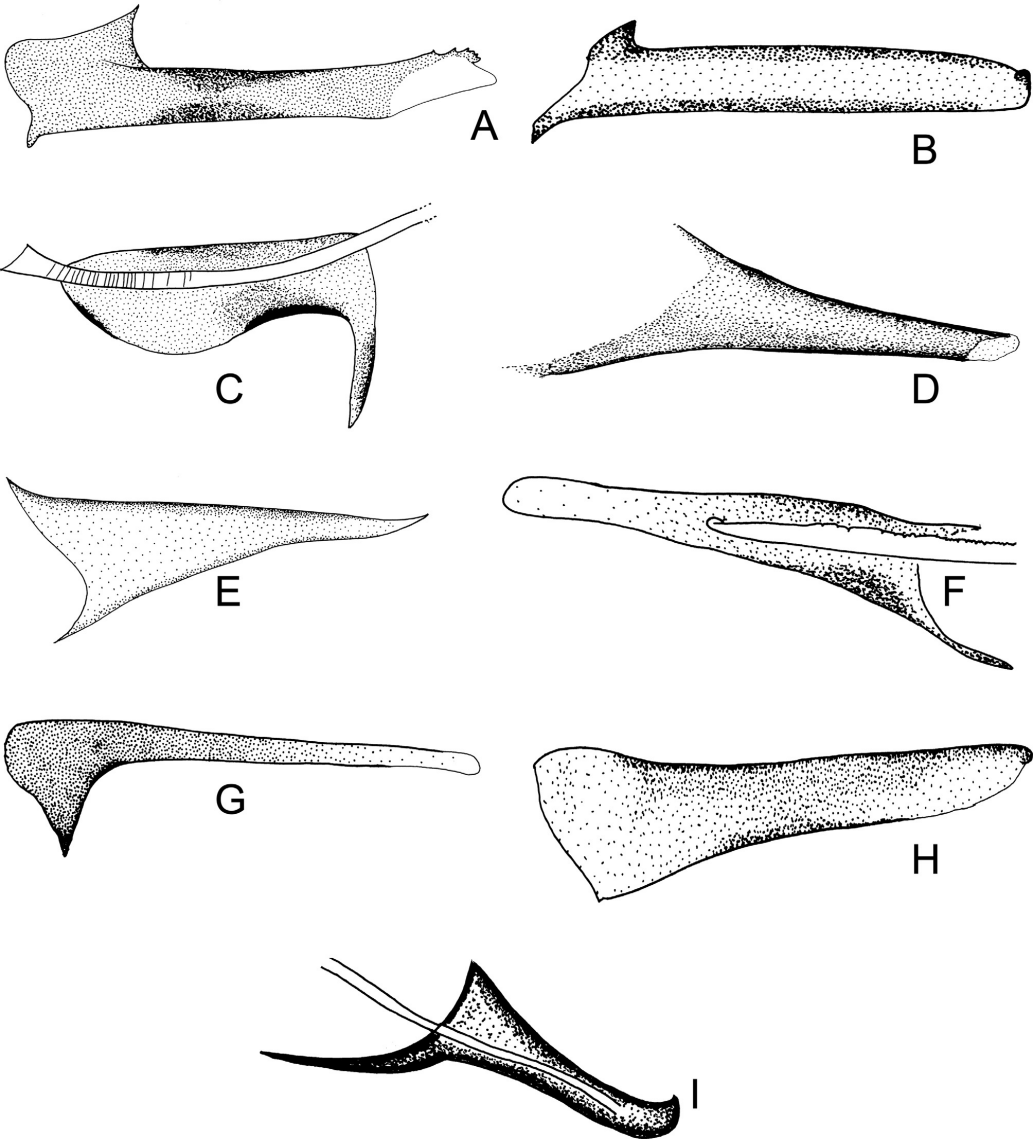
Parameral sheath of Phlebotominae. (A) With a transparent inferior top: *Phlebotomus perfiliewi*; (B) rounded with a knob at the top: *Idiophlebotomus nicolegerae*; (C) wide and short: *Chinius eunicegalatiae*; (D) with transparent top: *Phlebotomus* (*Euphlebotomus*) *barguesae*; (E) pointed: *Phlebotomus* (*Paraphlebotomus*) *chabaudi*; (F) rounded at the top: *Sergentomyia* (*Vattieromyia*) *anka*; (G) drumstick-like: *Phlebotomus* (*Larroussius*) *major*; (H) finger-like: *Phlebotomus* (*Madaphlebotomus*) *vaomalalae*; (I) with hooked top: *Phlebotomus* (*Paraphlebotomus*) *mongolensis*.

**Table 1.** Suggestion of the characters and the respective terminology used for the description of a new phlebotomine fly species. L = length; W = width; M = male; F = female; X = include the information.

Structures	Characters	Measurements	Description	Drawing
Head (MF)				
Occiput and clypeus	Distribution of the setae			X
Clypeus		L, W		X
Eyes		L, W		
Eye facets				X
Interantennal suture			X	X
Interocular sutures			X	X
Flagellomeres	f1, f2, f3, f12, f13, f14	L		X
	Position of the internal and external ascoids on f1		X	X
	Ascoidal formula		X	
	Number and position of papilla(e)		X	X
	Simple setae: position		X	X
Palpi	p1, p2, p3, p4, p5	L		X
	Palpal formula		X	
	p2, p3 – Newstead’s sensilla: presence and position/absence		X	X
	p3, p4: Numbers of spiniform setae		X	X
Labrum-epipharynx		L		X
Hypopharynx (F)	Apical teeth		X	X
Maxillary lacinia (F)	External and internal teeth: position		X	X
Labium (MF)	Labial suture: open or in furca		X	X
Cibarium (F)	Anterior teeth: horizontal/vertical/lateral position		X	X
	Posterior teeth: numbers		X	X
	Lateral teeth: presence/absence		X	X
	Sclerotized arch		X	X
	Sclerotized area		X	X
Pharynx (MF)	Teeth: presence/absence		X	X
Cervix (MF)				
Cervical sensilla	Number		X	
Ventro-cervical sensilla	Presence/absence		X	
Thorax (MF)		L		
Sclerites	Coloration		X	
Mesonotum	Post-alar setae: presence/absence		X	
Pleural sclerites	Paratergital setae: presence/absence		X	
	Proepimeral setae: presence/absence		X	
	Upper anepisiternal setae: presence/absence		X	
	Lower anapisternal setae: presence/absence		X	
	Anepimeral setae: presence/absence		X	
	Metaepisternal setae: presence (number)/absence		X	
	Metaepimeral setae: presence/absence		X	
	Setae on the anterior region of the katepisternum: presence/absence		X	
Suture between mesepimeron and metaepisternum	Presence/absence		X	
Metafurca: vertical arms	United/separated		X	
Wing	r5	L		
	Alpha, beta, gamma, delta, pi	L		
Legs	Coxa, femur, tibia, tarsomeres (ti, sum of tii, tiii, tiv, tv)	L		
	Metafemur-spines: presence/absence		X	
	Metatarsomere iii number of verticils with spines		X	
Abdomen		L		
Tergites ii–v (MF)	Setae forming or not two bands		X	
Tergites ii–vii (M)	Tergal papillae: presence/absence		X	
Genitalia (M)				
	Clusters of setae: presence and position/absence	L, W	X	X
	Sclerotized band in the ventral margin/presence/absence		X	X
Gonostyle		L		
	Spines: number, position, and aspects (size and thickness)		X	X
Parameres	Dorsal and ventral margin	L		
	Shape and covering bristle		X	X
Parameral sheath	Dorsal and ventral margin	L		
	Shape		X	X
Epandrial lobes		L, W		
	Permanent setae: presence/absence			X
Cerci; cercus (sing.)		L, W		X
Sperm pump		L, W	X	X
Ejaculatory apodeme		L, W	X	X
Aedeagal ducts		L	X	X
	Tip shape			
Hypandrial apodemes	Presence/absence		X	X
Genitalia (F)				
VIII tergite	Setae: presence (number)/absence		X	X
IX tergite	Protuberance: presence/absence		X	X
Spermathecae	Shape	L		
Terminal knob	Shape		X	X
Spermathecal individual ducts	Aspects: sclerotization, striation	L, W	X	X
Spermathecal common duct	Aspects: sclerotization, striation	L, W	X	X
Genital fork	Aspects of chamber and stem		X	X
Cerci; cercus (sing.)		L, W		
X sternite	Number of setae		X	X

**Table 2.** Suggested terminology for main characters for the description of a new phlebotomine fly species and the most common synonyms; French and Portuguese translations.

Suggested terminology	Synonyms	French	Portuguese
Head		Tête	Cabeça
Flagellomeres FI-FXIV	Antennomeres AIII–AXVI, antennal segments 3–16	Antennomères; segments antennaires a3–a16, articles antennaires AIII–AXVI	Antenômeros, segmentos antenais, artículos antenais
Ascoids	Geniculated spines	Ascoïdes; épines géniculées	Ascóides, espinhos geniculados
Antennal formula		Formule antennaire	Fórmula antenal
Papilla(e)	Hirsute glands; sensorial papillae	Sensilles	Papilas
Newstead’s sensilla	Newstead’s spines; Newstead’s sensory spines; Newstead’s modified spines; hyaline sensilla	Épines de Newstead, sensilles de Newstead	Espinhos de Newstead, sensilas de Newstead
Cibarium		Cibarium, cavité buccale	Cibário,cavidade bucal
Cibarial armature		Armature cibariale	Armadura cibarial
Posterior teeth	Horizontal teeth	Dents cibariales	Dentes horizontais, dentes posteriores
Anterior teeth	Vertical teeth	Denticules	Dentes verticais, dentes anteriores
Sclerotized arch	Chitinous arch	Arc chitineux	Arco esclerosado, arco esclerotinizado, arco quitinoso.
Sclerotized area	Pigmented area; pigment patch	Plage pigmentée	Área esclerotinizada, área esclerosada, área pigmentada, área quitinizada.
Pharyngeal teeth	Spines of pharynx	Dents pharyngéeS	Espinhos da faringe
			
Thorax		Thorax	Tórax
Post-alar seta(e)		Soies rétro-alaires	Cerdas pós-alares
Sclerites		Sclérites	Escleritos
Proepimeron		Proépimère	Proepimero
Proepimeral setae	Lower anepisternal setae	Soies mésanepistérnales inférieures	Cerdas proepimerais, cerdas anepisternais inferiores
Anepisternum	Mesanepisternum	Mésanepisterne	Anepisterno
Upper anepisternal setae		Soies mésanepistérnales supérieures	Cerdas anepisternais superiores
Lower anapisternal setae		Soies mésanepistérnales inférieures	Cerdas anepisternais inferiores
			
Abdomen		Abdomen	Abdômen, Abdome
Trumpet glands		Glandes en trompettes	
Tergal papillae			Papilas tergais
			
Genitalia (M)	Hypopygium	Genitalia, hypopygium	Genitália, hipopígio
Gonocoxite	Basimere, basistyle; coxite; proximal segment of upper gonapophyse; basal segment of upper appendages; superior clasper	Coxite; gonocoxite; gonapophyse supérieure	Basistilo, gonocoxito, coxito, segmento proximal da gonapófise superior, segmento basal do apêndice superior, claspete superior
Gonostyle	Distimere, distal segment of upper gonapophyses; terminal segment of upper appendages; terminal segment of clasper; style	Style, gonostyle	Dististilo, gonóstilo
Parameres	Intermediate appendages; intermediate gonapophyses; claspers; claspette, middle appendages;	Paramère; appendice intermédiaire	Parâmeros, apêndices intermediários, gonapófise, claspetes, apêndices medianos
Cerci; cercus (sing.)		Cerques	Cercos
Epandrial lobes	Cerci, lateral lobes; lateral lobe of IX tergite, lower gonapophyses, surstyle	Cerques, lobes latéraux; surstyles	Lobos laterais do ixº tergito, surstilo, gonapófise inferior
Sperm pump	Genital pump; ejaculatory pump; pompetta	Pompe génitale	Bomba ejaculadora, pompeta
Ejaculatory apodeme	Piston; rod	Piston	Pistão
Sperm sac	Sac of pompetta, seminal pump	Pavillon de la pompe	Câmara ejaculadora
Aedeagal ducts	Aedeagal filaments; aedeagus; ejaculatory ducts; ejaculatory filaments genital filaments; spicules; intromitent organ, penal filaments	Filaments génitaux; canaux éjaculateurs; organes intromittents; filaments péniens	Espículos, filamentos penianos, filamentos ejaculadores, dutos ejaculadores, órgão intromitente
Parameral sheath	Aedeagal sheath; aedeagus; gubernaculum; penial valve; penis, penal theca	Édéage, fourreau pénien; gaine du pénis; pénis; valve pénienne.	Edeago, pênis, gubernáculo, valva peniana, teca peniana
Hypandrial apodemes	Gonocoxal hypandrial apodeme of the aedeagal sheath; sclerotized rods	Baguettes abdominales	Hastes esclerosadas
			
Genitalia (F)			
Spermatheca	Body of spermatheca	Spermathèque; corps de la spermathèque	Corpo da espermateca
Terminal knob	Head of the spermatheca	Tête de la spermathèque	Cabeça da espermateca
Spermathecal ducts	Ducts	Conduits des spermathèques	Dutos das espermatecas
Genital fork	Genital furca	Furca génitale	Furca genital

Lastly, an update to the previous proposal for abbreviating the names of genera and subgenera [8] is presented in Table 3.

**Table 3.** Proposed abbreviations for genera and subgenera of Phlebotominae.

Genus	Abbreviation	Subgenus	Abbreviation
*Australophlebotomus* Theodor	*Au.*		
*Brumptomyia* França & Parrot	*Br.*		
*Chinius* Leng			
*Dampfomyia* Addis	*Da.*		
		*Coromyia* Barretto	*Cor.*
		*Dampfomyia*	*Dam.*
*Deanemyia* Galati	*De.*		
*Edentomyia* Galati, Andrade Filho, Silva & Falcão	*Ed.*		
*Evandromyia* Mangabeira	*Ev.*		
		*Aldamyia* Galati	*Ald.*
		*Barrettomyia* Martins & Silva	*Bar.*
		*Evandromyia*	*Eva.*
*Expapillata* Galati	*Ex.*		
*Grassomyia* Theodor	*Gr.*		
*Hertigia* Fairchild	*He.*		
*Idiophlebotomus* Quate & Fairchild	*Id.*		
† *Libanophlebotomus* Azar	*Lb.*		
*Lutzomyia* França	*Lu.*		
		*Castromyia* Mangabeira	*Cas.*
		*Lutzomyia*	*Lut.*
		*Helcocyrtomyia* Barretto	*Hel.*
		*Tricholateralis* Galati	*Trl.*
*Martinsmyia* Galati	*Mt.*		
† *Mesophlebotomites* Azar	*Me.*		
*Micropygomyia* Barretto	*Mi.*		
		*Coquillettimyia* Galati	*Col.*
		*Micropygomyia*	*Mic.*
		*Sauromyia* Artemiev	*Sau.*
		*Silvamyia* Galati	*Sil.*
*Migonemyia* Galati	*Mg.*		
		*Blancasmyia* Galati	*Bla.*
		*Migomemyia*	*Mig.*
*Nyssomyia* Barretto	*Ny.*		
*Oligodontomyia* Galati	*Ol.*		
† *Palaeomyia* Poinar Jr,	*Pl.*		
*Parvidens* Theodor & Mesghali	*Pv.*		
† *Phlebotoiella* Solórzano Kraemer & Wagner	*Pb.*		
† *Phlebotomiella* Hennig	*Pe.*		
† *Phlebotomites* Meunier	*Pt.*		
*Phlebotomus* Rondani & Berté	*Ph.*		
		*Abonnencius* Morillas Marquez, Castillo Remiro & Ubeda Ontiveros	*Abo.*
		*Adlerius* Nitzulescu	*Adl.*
		*Anaphlebotomus* Theodor 1948	*Ana.*
		*Euphlebotomus* Theodor, 1948	*Eup.*
		*Kasaulius* Lewis	*Kas.*
		*Larroussius* Nitzulescu	*Lar.*
		*Legeromyia* Rahola, Depaquit, Makanga & Paupy	*Leg.*
		*Madaphlebotomus* Depaquit, Leger, Randrianambinintsoa	*Mad.*
		*Paraphlebotomus* Theodor	*Par.*
		*Phlebotomus*	*Phl.*
		*Synphlebotomus* Theodor	*Syn.*
		*Transphlebotomus* Artemiev	*Tra.*
*Pintomyia* Costa Lima	*Pi.*		
		*Pifanomyia* Ortiz & Scorza	*Pif.*
		*Pintomyia*	*Pin.*
*Pressatia* Mangabeira	*Pr.*		
*Psathyromyia*	*Pa.*		
		*Forattiniella* Vargas	*For.*
		*Psathyromyia*	*Psa.*
		*Xiphopsathyromyia* Ibánez-Bernal & Marina	*Xip.*
*Psychodopygus* Mangabeira	*Ps.*		
*Sciopemyia* Barretto	*Sc.*		
*Sergentomyia* França & Parrot	*Se.*		
		*Capensomyia* Davidson, 1979	*Cap.*
		*Demeillonius* Davidson	*Dem.*
		*Grassomyia* *	*Gra.*
		*Neophlebotomus* França & Parrot	*Neo.*
		*Parrotomyia* Theodor	*Par.*
		*Rondanomyia* Theodor	*Ron.*
		*Sergentomyia*	*Ser.*
		*Sintonius* Nitzulescu	*Sin.*
		*Trouilletomyia* Depaquit, Léger & Randrianambinintsoa	*Tro.*
		*Vattieromyia* Depaquit, Léger & Robert	*Vat.*
*Spelaeomyia* Theodor	*Sa.*		
*Spelaeophlebotomus* Theodor	*Sl.*		
*Trichophoromyia* Barretto	*Th.*		
*Trichopygomyia* Barretto	*Ty.*		
*Viannamyia* Mangabeira	*Vi.*		
*Warileya* Hertig	*Wa.*		

* If considered as a subgenus;

† Fossil.

## Other taxonomic approaches

It is becoming more straightforward to distinguish phlebotomine taxa using modern techniques. The use of statistical approaches, such as models based on discriminant or multivariate analyses used in morphometric studies, may also contribute to the identification of intra- and inter-specific differences.

Concerning the identification of species using methods other than traditional dichotomous keys, the development of cybertaxonomy may facilitate this task for non-specialists or when many specimens need to be identified. The limited number of characters used by this tool may restrict its application in the case of a diverse fauna on a large scale.

One of the significant challenges facing phlebotomine taxonomists is the adoption of an integrative taxonomic approach, with an increase in attention to identifying characters to be used for accurate and efficient delimitation of species. Well-known, common adult morphological and morphometric characters are important; however, others such as behavioral, biochemical, ecological, and molecular data need to be considered, as well as morphological or developmental characters related to eggs, larvae, and pupae. It is highly recommended that markers used in molecular analysis for the delimitation of taxa be standardized and gene sequences should be deposited in free-access databases to permit the analysis of species or populations, especially for widespread taxa. This information is also valuable for phylogenetic studies.

The taxonomy of American phlebotomines has recently been updated regarding the number of described species/subspecies (502: 17 fossil and 485 extant). Additionally, it was commented that there has been an increase of 16.5% in the number of species described since Galati’s classification [3] and of 22.5% since that of Young and Duncan [11], though, in this latter case, only for the groups that these authors included in *Lutzomyia*. It was also commented that some taxa had been resurrected, some other species had been included as junior synonyms and two artificial taxa had been excluded from the species list. It is important to note that Galati’s classification has been updated annually and is available on the website: www.fsp.usp.br/~egalati [5].

Finally, the need to strengthen the training of taxonomist groups around the world was emphasized in view of the fact that taxonomy is the basis of the eco-epidemiological studies of vector-borne diseases. In recent years, with the advances of new technologies, particularly the molecular approach, a low level of interest on the part of young researchers in classical taxonomy has been observed that could lead to a significant loss of knowledge because expertise in this field depends on the strength of training and mentorship over successive generations.
